# Human Left Ventricle circRNA-miRNA-mRNA Network Analyses Reveal a Novel Proangiogenic Role for circNPHP1 Under Ischemic Conditions

**DOI:** 10.1016/j.jacbts.2025.101468

**Published:** 2026-01-20

**Authors:** Maryam Anwar, Moumita Sarkar, Kerrie Ford, Gianni D. Angelini, Prakash P. Punjabi, Yingshu Guan, Abas Laftah, Aránzazu Chamorro-Jorganes, Jiahui Ji, Prashant K. Srivastava, Enrico Petretto, Costanza Emanueli

**Affiliations:** a National Heart and Lung Institute, Imperial College London, London, United Kingdom; b Bristol Heart Institute, University of Bristol, Bristol, United Kingdom; c Hammersmith Hospital, Imperial College Healthcare National Health Service Trust, London, United Kingdom; d Centre for Computational Biology, Duke-NUS Medical School, Singapore

**Keywords:** angiogenesis, bioinformatics, diabetes, endothelial cells, Human studies, ischemic heart disease, noncoding RNAs, transcriptomics

## Abstract

Therapies promoting microvascular flow and endothelial repair require effective identification of new targets in ischemic heart disease (IHD). The aim of this study was to unravel and functionally investigate circular RNA (circRNA)-microRNA (miRNA)-messenger RNA (mRNA) networks in endothelial cells in the context of IHD and type 2 diabetes mellitus (T2DM). We performed RNA sequencing on left ventricle biopsies from patients with IHD, IHD and T2DM, and non-IHD. Next, we created circRNA-miRNA-mRNA networks and mechanistically investigated the top circRNA-miRNA sponging interactions in endothelial cells. We found that circNPHP1 promotes angiogenesis via sponging of miR-221-3p and regulates its downstream target genes, VEGFA and BCL2, in IHD and T2DM.

## Highlights

• We found a novel circRNA-miRNA-mRNA network in IHD and T2DM.
• CircNPHP1 regulates angiogenesis and proliferation in the cardiac endothelial cells exposed to conditions mimicking IHD and T2DM.
• We elucidated a novel proangiogenic subnetwork consisting of circNPHP1, miR-221-3p, BCL2, and VEGFA.
• We identified circNPHP1 as a potential new target for therapeutic angiogenesis.

## Visual Abstract

**Figure:**
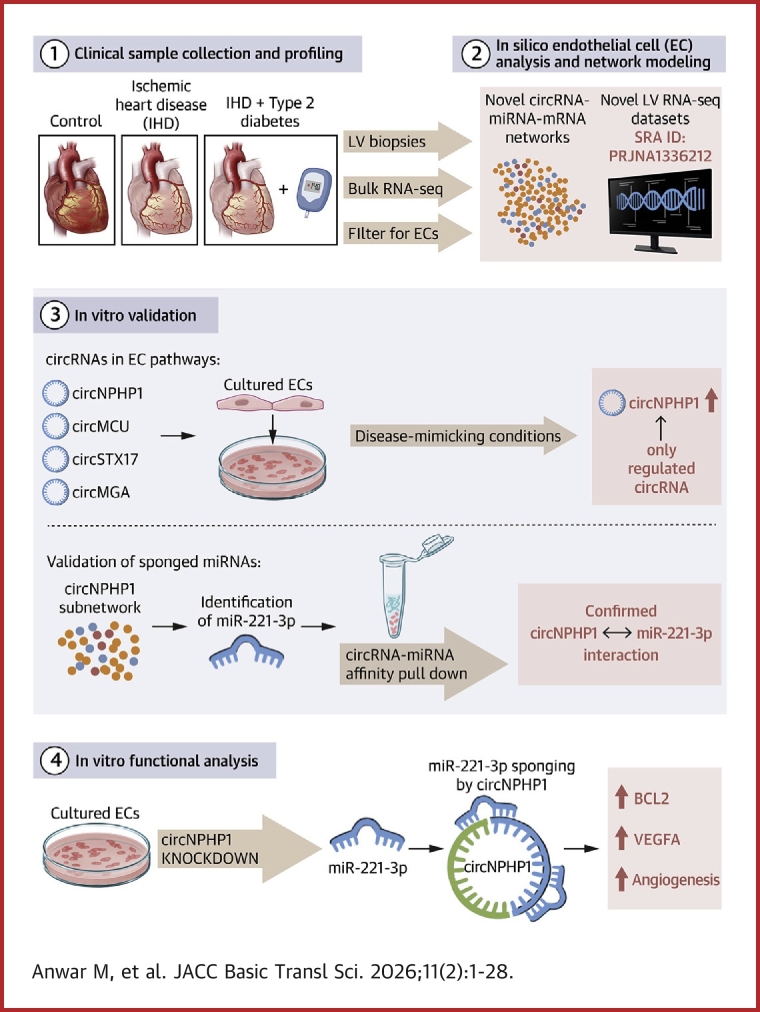

Ischemic heart disease (IHD) can trigger adverse cardiac remodeling leading to heart failure and remains a major cause of premature death worldwide.^1^ Coronary artery disease (CAD) is the prevalent cause of IHD. Diabetes aggravates CAD and impinges microangiopathy, further compromising the coronary artery and microvascular blood flow.^2^ Additionally, diabetes impairs the potential for compensatory myocardial angiogenesis.^3^ A prime target of hyperglycemia is the vascular endothelial cells (ECs), which line the blood vessels lumen. Endothelial dysfunction significantly contributes to the pathophysiology of diabetes’ macrovascular and microvascular complications.^4^^,^^5^ Conversely, ECs play a pivotal role in reparative angiogenesis and in maintaining the cardiac functions.^6^^,^^7^ The identification and validation of novel molecular mechanisms that regulate the endothelium of the diabetic heart is of great pathophysiological significance and could additionally aid in the repurposing and design of therapeutics. Indeed, RNA therapeutics are currently in the spotlight for treatment of atherosclerosis cardiovascular disease^8^ and heart failure.^9^

CircRNAs are single-stranded, covalently bonded loop structures.^10^ Their circular shape makes them resistant to exonucleases and hence more stable than other RNA forms.^11^ CircRNAs, which were first reported as expressed in humans in 1993, were initially considered as products of “mis-splicing.”^12^ Later studies indicated their role as potential regulators of physiological functions.^13^^,^^14^ CircRNAs have been implicated in post-transcriptional regulation of gene expression through the “sponging” of microRNAs (miRNAs).^15^ miRNAs are small noncoding RNAs, which can become a part of RNA-induced silencing complex to repress messenger RNAs (mRNAs). Canonically, an miRNA recognizes its mRNA targets using its miRNA seed sequence, which is semicomplementary to 1 or more binding sites usually placed in the 3’-UTR of the mRNAs.^16^^,^^17^ CircRNAs bind to miRNAs via multiple miRNA response elements (MREs) which make the miRNAs unavailable to bind to and repress their target mRNAs.^18^ This results in the derepression of the miRNA target gene, increasing the target gene’s mRNA and protein levels.

In contrast to linear RNA, circRNAs are generated through back splicing that connects the exon toward the 3′ end to one of the exons close to the 5′ end, thus forming a circular structure.^10^ This alteration in exon order provides a criterion for efficiently detecting back-spliced junctions during the alignment step in bioinformatics analyses.^14^

We recently reviewed the RNA regulatory role of circRNAs in IHD and cardiac remodeling.^19^^,^^20^ CircRNAs have been reported to regulate cardiac angiogenesis^21^ and atherosclerosis consequent to flow-dependent endothelial inflammatory responses.^21^, ^22^, ^23^ in mice. Made et al^24^ recently investigated circRNA-miRNA-mRNA circuits in the human failing hearts using samples collected during both the surgical ventricular reconstruction procedure and heart transplantation. However, the circRNAs governing endothelial-relevant miRNA-mRNA networks in the pre–heart failure left ventricle (LV) affected by IHD and T2DM remains largely unexplored. This is significant because endothelium-targeted intervention is expected to have more value at this stage of disease progression where they could be synchronized with surgical coronary revascularization.

To characterize clinically relevant circRNAs, miRNAs, and mRNAs, our novel study has employed a reverse translational approach based on the use of LV biopsies collected from female and male IHD patients with/without T2DM during a coronary artery bypass graft surgery procedure. The nonischemic, nondiabetic control group was formed by donors undergoing mitral valve repair as the sole procedure. The mitral valve repair patients were not affected by angina, heart failure, arrhythmia, or any infection and immunity conditions. Further details are provided in the Methods, when describing the ARCADIA (Association of Non-coding RNAs With Coronary Artery Disease and Type 2 Diabetes) study, which also included the predefinition of the ARCADIA analyses plans. We have applied an integrative approach involving bioinformatics and experimental validations in the heart samples and cultured cardiac cell models.

## Methods

### ARCADIA sample collection

ARCADIA (REC Number: 13/LO/1687) is a prospective observational clinical study developed between 2 centers: the Bristol Royal Infirmary (University Hospitals Bristol NHS Foundation Trust) and the Hammersmith Hospital (Imperial College Healthcare NHS Trust). Written informed consent was obtained from all patients. All human samples were obtained in accordance with the principles of the Declaration of Helsinki. The study was reviewed and approved by the National Research Ethics Service Committee London–Fulham (date of approval, December 20, 2013; reference REC 13/LO/1687), which also received our voluntary submission of the ARCADIA stage 1 analysis plan (Supplemental File 1). A second cohort of patients was also recruited as part of the ARCADIA study for validation experiments by targeted molecular analyses. Patients’ characteristics of cohorts 1 and 2 are presented in Supplemental Tables 1 and 2, respectively.

### Clinical sample collection and processing

LV biopsies were taken from the apex of the heart using a biopsy needle. Detailed methods are available in Ford et al,^25^ a methodological paper, which reports our optimization of the protocol for RNA extraction, rRNA depletion, libraries preparation, and RNA sequencing (RNA-seq) (Illumina HiSeq 2500 and Qiagen-small RNA-seq). Peripheral blood samples were collected in citrate buffer at different time points around cardiac surgery. For this study, we limited the analysis to circRNA quantitative real-time polymerase chain reaction (qRT-PCR) (see the following text) on the sample collected in the anesthesia room, before cardiac surgery initiation.

### Bioinformatics analyses of RNA-seq data from LV biopsies

Two data sets were generated from bulk RNA-seq: Whole transcriptome and small RNA from LV biopsies. The quality of sequenced data was assessed using the program FASTQC.^26^ The library size for small RNA (LV biopsies) was estimated at 20 million reads (read length of 50-60 base pairs [bp]) and whole transcriptome (biopsies) was estimated at 70 million paired-end reads (read length of 100 bp). Samples were next aligned to human genome (hg19) using the aligner STAR.^27^ Raw read counts were generated using the R package Rsubread (R Foundation for Statistical Computing),^28^^,^^29^ and annotations were carried out using Ensembl GRCh37 version 75 (whole transcriptome) and miRBase version 20 (small RNA). Raw read counts were normalized using the Trimmed Mean of M-values method, and differential expression (DE) was calculated between the different groups using the R package *Limma.*^30^ CircRNAs were detected and annotated using CIRCexplorer.^31^ CircRNAs were considered expressed in a particular group if at least 50% of the samples within that group had nonzero expression values for the circRNA. This was followed by normalization (fragments per million) and DE analyses (R package *Limma*). Fold changes and *P* values were generated using *Limma*. False discovery rate (FDR) was used as a post hoc test (also generated using *Limma*). In line with an inclusive and exploratory approach, a *P* value threshold of <0.05 was used for finding DE circRNAs, miRNAs, and mRNAs.

### Prediction of circRNA-miRNA-mRNA networks present in the human LV

**Figure 1:**
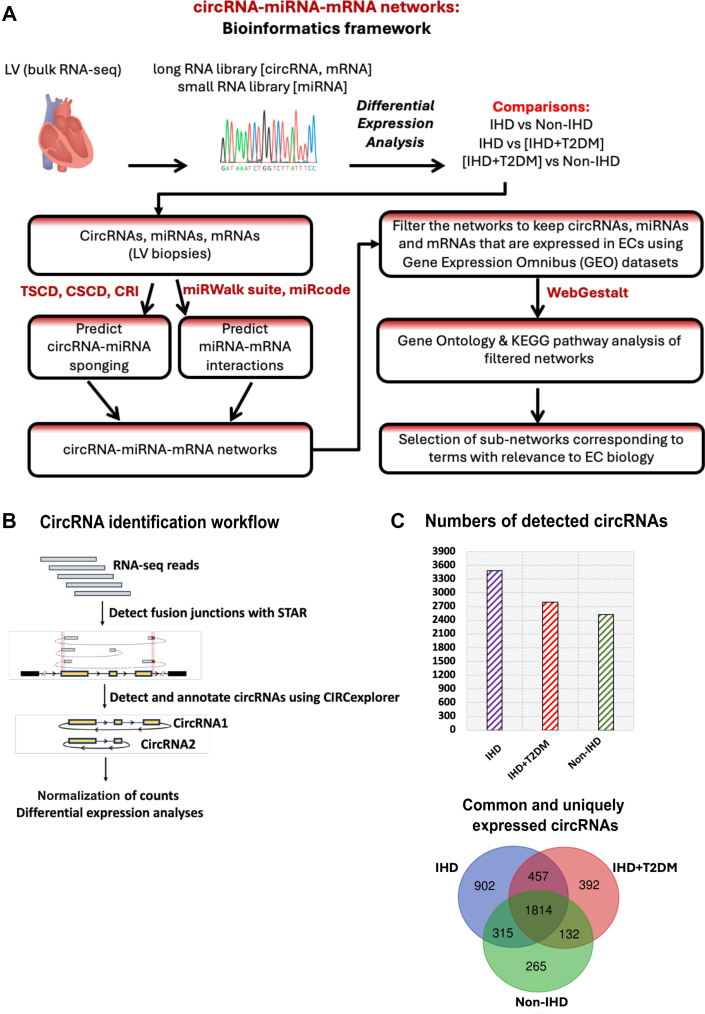
Identification and Quantification of circRNAs (A) A bioinformatics framework to build circular RNA (circRNA)-microRNA (miRNA)-messenger RNA (mRNA) networks: after extracting RNAs (circRNAs, miRNAs, and mRNAs) from RNA sequencing (RNA-seq) data on left ventricle (LV) biopsies of cardiac surgery patients (ischemic heart disease [IHD] [n = 12], IHD+ type 2 diabetes mellitus [T2DM] [n = 11], non-IHD [n = 12]), differential expression analyses were performed. Next, the circRNA-miRNA sponging interactions were predicted using databases: Tissue-Specific circRNA Database (TSCD) (http://gb.whu.edu.cn/TSCD/), cancer-Specific circRNA Database (CSCD) (http://gb.whu.edu.cn/CSCD), and Circular RNA Interactome (CRI) (https://circinteractome.irp.nia.nih.gov/). The miRNA-mRNA interactions were predicted using miRWalk (http://mirwalk.umm.uni-heidelberg.de/) and miRcode (https://bio.tools/miRcode). Furthermore, only those interactions were kept where the connecting nodes (circRNAs, miRNA, and mRNAs) were expressed in the endothelial cells (ECs) (using published EC data sets from GEO). Gene Ontology and Kyoto Encyclopedia of Genes and Genomes (KEGG) pathway analyses were subsequently performed on these filtered networks. Sections of the network that mapped to processes corresponding to EC biology were further analyzed. (B) *CircRNA identification workflow:* RNAseq reads from whole transcriptome were first aligned using STAR aligner, then circRNAs were identified using CIRCexplorer with annotations from CircBase. Following that, raw counts were normalized to fragments per million, and differential analyses was carried out using R package *Limma.* (C) Bar graph showing number of detected circRNAs in IHD, IHD+T2DM and non-IHD. CircRNAs that had nonzero expression values in at least 50% of the samples of a patient group were considered expressed/detected in that particular group. Venn diagram shows common and uniquely expressed/detected circRNAs in the 3 patient groups: IHD (n = 12), IHD+T2DM (n = 11), non-IHD (n = 12).

We predicted sponging interactions between circRNAs (LV biopsies) and miRNAs using different tools/databases: TSCD (Tissue specific CircRNA database),^32^ CSCD (Cancer-Specific CircRNA Database),^33^ and CRI (Circular RNA Interactome).^34^ Binding confirmation of miR-221-3p to back-splice junction sequence of circNPHP1 was predicted using Circbank.^35^ We predicted miRNA–mRNA interactions using miRWalk suite^36^ and miRcode.^37^ All interactions passing the threshold of binding probability (>0.95) were retained. Following this, we created circRNA-miRNA-mRNA networks corresponding to IHD and IHD+T2DM in Cytoscape.^38^ We extracted all circRNAs, miRNAs, and mRNAs from the networks that were expressed in human ECs based on Gene Expression Omnibus RNA-seq data sets: GSE100242^39^ (circRNAs, human umbilical artery endothelial cells), GSE53315^40^ (miRNAs, human coronary artery endothelial cells), and GSE134489^41^ (mRNAs, human coronary artery endothelial cells). Gene ontology (GO) and KEGG (Kyoto Encyclopedia of Genes and Genomes) pathway analysis was carried out using WebGestalt. Whereas GO provides information on cellular compartmentalization, biological processes, and molecular function of genes, KEGG is a collection of manually drawn pathways representing information of the molecular interaction between genes and reactions for metabolic processes. KEGG pathways and GO biological processes that were significant with an FDR <0.05 were reported. Following pathway analysis, each of the 3 networks were scanned to identify circRNAs, miRNAs, and mRNAs that mapped to EC-relevant pathways. From these, circRNAs that mapped to EC-relevant pathways were selected for expressional analyses in cultured ECs. The miRNA and mRNA partners of circRNAs (that showed significant differences in cultured ECs under disease-mimicking conditions) were further investigated. A pipeline indicating these steps is given in Figure 1A.

### Single-cell and single-nuclei RNA-seq data analysis

The raw data for single-nuclei and single-cell RNA-seq from healthy donor hearts were acquired from the human heart atlas.^42^ The pseudo counts were calculated as the sum of Unique Molecular Identifiers (UMIs) in cells of each cell type from 1 donor and further normalized as counts per million. Boxplots were created to show the expression across different cell types, presented as log2-transformed counts per million values.

### RNA isolation and qRT-PCR in tissue samples

Total RNA was isolated from the tissue samples using QIAzol reagent (Qiagen) and mirVana kit (Thermo Fisher Scientific) following manufacturer’s protocol. For detection of circRNAs, divergent primers against the back-splice junction sequence were designed using NCBI Primer Design webtool. CircBase ID for circRNA candidates was identified according to the specific genomic coordinates. A 200-nt long junction sequence, combining 100 nt from the 3′ end of the circRNA sequence to 100 nt from the 5′ end, was acquired using the Circular RNA Interactome webtool as the PCR amplicon sequence used for divergent primer design. For the analysis of circRNAs, cDNA was synthesized using the PrimeScript qRT-PCR kit (Takara), according to the manufacturer’s instructions. qRT-PCR was performed using TB Green Premix Ex Taq Kit (Takara) on QuantStudio 6 Flex Real-Time PCR System (Life Technologies). 18S rRNA (ribosomal RNA) was used for normalization of circRNAs in the biopsies. All of the PCR primer sequences are listed in the resources table (Supplemental File 3).

### RNA isolation and qRT-PCR in patient plasma samples

For the quantification of circNPHP1 and linear NPHP1 in the patients’ plasma samples, RNA was extracted from 200-μL plasma using QIAzol and the miRNeasy Serum/Plasma Kit (Qiagen) according to the manufacturer’s instructions. 1 femtomole of external spike-in cel-miR-39 (Norgen Biotek) was added to the plasma samples during RNA extraction. As mentioned in the previous text, cDNA was synthesized using the PrimeScript RT-PCR kit, and qRT-PCR was performed using TB Green Premix Ex Taq Kit on QuantStudio 6 Flex Real-Time PCR System (Life Technologies). GAPDH was used as endogenous control and cel-miR-39 was used as spike-in control for normalization of circRNAs. All of the PCR primer sequences are listed in the resources table (Supplemental File 3).

### Cell culture

Human cardiac microvascular endothelial cells (HCMECs) (Promocell: C-12285 [mono-doner], Lot numbers: 440Z021.5 [male], 440Z021.4 [male], 446Z001.1 [female], 492Z009.4 [female]) and human umbilical vein endothelial cells (HUVECs) (Promocell: C-12208 [poly-donor], Lot numbers: 447Z004, 450Z015, 466Z022, 503Z026) were cultured on 0.2% gelatin (Sigma-Aldrich) in endothelial cell growth medium MV and endothelial cell growth medium 2 (EGM2) (PromoCell). The biological replicates of experiments done on the ECs were carried out on cells from different lots. Human cardiac fibroblasts (Promocell: C-12375; Lot number:491Z026.1) were cultured in fibroblast growth medium 3 (PromoCell). Proliferating AC16 human cardiomyocytes (Sigma-Aldrich SCC109, a gift from Professor Rajesh Katare, Otago University) were maintained in basal media (DMEM/F-12 with 10% fetal bovine serum, 1% L-glutamine, and 1% penicillin/streptomycin). The biological replicates in AC16 and cardiac fibroblasts were carried out on cells from different passages of the same lot. Cell cultures were maintained at 37 °C in a humidified atmosphere containing 5% CO_2_. For mimicking ischemic conditions, cells were exposed to hypoxia (1% O_2_ in a humidified atmosphere containing 5% CO_2_) for 48 hours at 37 °C. For mimicking diabetes hyperglycemia, cells were cultured in 25 mmol/L D-glucose (high glucose [HG]). Cells were cultured in hypoxia and HG to mimic the association of IHD and diabetes. 25 mmol/L L-glucose (Mannitol) was used as an osmotic control. Cells were counted using standard hemocytometer using Trypan blue stain.

### RNA-seq of cells and bioinformatics analyses

For bulk whole transcriptome sequencing of different cell types, 1 μg of RNA from HCMECs, HUVECs, cardiomyocytes (AC16), and cardiac fibroblasts were sent to Biomarker Technologies (BMKGENE UK LTD) for library preparation and RNA-seq. The library size for whole transcriptome on cells was estimated at 60 million paired-end reads (read length of 150 bp). Samples were processed and analyzed in the same way as given in the Methods, section “Bioinformatics Analyses of RNA-seq Data from LV Biopsies,” for the detection of circRNAs and mRNAs in cells.

### RNase digestion assay

A total of 2 μg of RNA from HUVEC was incubated with 10 U RNase R (New England Biolabs) and 10 U of RiboLock RNase Inhibitor (Thermo Fisher Scientific) in 1× RNase R buffer in a 10 μL reaction at 37 °C for 30 minutes, followed by heat inactivation at 95 °C for 3 minutes. Subsequently, the RNA was purified using RNA Clean and Concentrator™-5 kit (Zymo Research) following manufacturer’s instructions.

### Transfection of oligonucleotides

HCMECs and HUVECs were seeded (1 × 10^4^ cells/well) in a 96-well plate and transfected with 30 nmol/L of circNPHP1 siRNA (custom siRNA1 or siRNA2), siRNA control (nontargeting negative control pool) (Horizon Discovery), or linear NPHP1 (Santa Cruz Biotechnology). The specificity of the siRNAs were validated combining the use of circBase BLAT (BLAST-like alignment tool) to query the circBase database, which encompasses all circular RNAs and NCBI BLAST (Basic Local Alignment Search Tool) against the Nucleotide Collection Database, which comprises all linear (coding and noncoding) RNAs. Likewise, 30 nmol/L of hsa-miR-221-3p mimic or miRNA mimic negative control (Horizon Discovery).; anti-hsa-miR-221-3p or anti-miRNA negative control (Thermo Fisher Scientific) were transfected in the ECs.

AC16 and cardiac fibroblasts were seeded (1 × 10^4^ cells/well) in a 96-well plate and transfected with circNPHP1 siRNA (30 nmol/L) or control siRNA. All of the transfections were carried out using Lipofectamine 2000 (Thermo Fisher Scientific) as described.^43^ Details of the sequences and catalogues are provided in the resources table (Supplemental File 3). At 48 hours post-transfection, cells were used for experiments described in the following subsections.

### RNA isolation and qRT-PCR in the cells

Total RNA was isolated from the cells using QIAzol reagent (Qiagen) and miRNeasy Mini Kit (Qiagen) following manufacturer’s protocol. For detection of circRNAs, divergent primers were used as mentioned earlier. For the analysis of circRNAs and mRNAs, cDNA was synthesized using the PrimeScript RT-PCR kit (Takara), and qRT-PCR was performed using TB Green Premix Ex Taq Kit (Takara) following manufacturer’s instructions. 18S rRNA (ribosomal RNA) was used for normalization of circRNAs and mRNAs in the cells. For the quantification of miRNAs, cDNA was synthesized using miRCURY LNA RT Kit (Qiagen). qRT-PCR was performed using miRCURY LNA SYBR Green PCR Kit (Qiagen) following manufacturer’s instructions. Small RNA U6 was used for normalization of miRNAs. All of the qRT-PCRs were performed on QuantStudio 6 Flex Real-Time PCR System (Life Technologies). All of the PCR primer sequences are listed in the resources table (Supplemental File 3).

### CircRNA pulldown

The circNPHP1 pulldown was carried out in HUVECs, cardiac fibroblasts, and AC16-cardiomyocytes according to the protocol described by Das et al.^44^ The circRNA was pulled down using antisense-oligo (ASO) probe synthesized with biotin-TEG added to the 3′ end of the sequences (Sigma-Aldrich). The first probe was designed by joining the last 15 nucleotides of circNPHP1 to the first 15 nucleotides to make a 30-nucleotide sequence against the back-splice junction followed by reverse complement. The second probe (probe 2) used to pulldown circNPHP1 consists of a 22-nucleotide sequence targeting the unique back-splice junction of circNPHP1 (see Supplemental Figure 1A), which is the same sequence we targeted for designing the siRNA for circNPHP1 used in this study. The specificity of the 2 ASO probes were validated combining circBase BLAT and NCBI BLAST analysis. Control ASO was selected according to the protocol.^44^ The sequences of the probes are listed in the resources table (Supplemental File 3) and additionally indicated in Supplemental Figure 1A.

The brief protocol for pulling down the circNPHP1 was the following. For each cell type, 5 million cells were harvested in Tris, KCl, MgCl_2,_ nondiet P-40-polysome extraction buffer. Cell extracts were incubated with 1 μL of the biotinylated probes or the control probe (100 μmol/L) in Tris, EDTA, NaCl, Triton (TENT) buffer with rotation for 90 minutes at 4 °C for hybridization. Subsequently, streptavidin beads (washed in TENT buffer) were added and the mixture of the cell extract, probes, and the beads was incubated with rotation for further 45 minutes at room temperature. Then, the beads were washed thrice in TENT buffer followed by isolation of RNA using Trizol and phenol-chloroform. cDNA was synthesized and subjected to qRT-PCR for determination of circNPHP1 and the bound miRNAs. Fold enrichment was calculated in the pulldown samples with probes 1 and 2 compared with the control probe using the delta delta Ct method (Livak). 18S was used as reference gene to normalize for circNPHP1 and linear NPHP1 and U6 was used as to normalize for the miRNAs. In HUVECS, 4 miRNAs were screened namely miR-221-3p, miR-222-3p, miR-299-3p, and miR-141-3p. Subsequently, in the pulldown assays performed in cardiac fibroblasts and AC16, miR-221-3p was checked for binding to circNPHP1.

### miRNA pulldown

The miRNA pulldown was carried out in HUVECs according to the protocol described by Phatak and Donahue.^45^ The miRNAs were pulled down using custom synthesized miRNAs (miRNA-221-3p, miRNA-222-3p, and miRNA-139-5p) with biotin added to the 3′ end of the sequences (Sigma-Aldrich). Control miRNA sequence was chosen following the protocol. All of the sequences are listed in the resources table (Supplemental File 3). The brief protocol for pulling down the miRNAs was as follows. Biotinylated miRNAs or the control miRNA were transfected in a 10-cm tissue culture dish, respectively. At 48 hours post-transfection, cells were harvested in lysis buffer (containing Tris-HCl, KCl, MgCl_2_, and IGEPAL CA-630). Cell extracts were incubated with streptavidin dyna-beads overnight with rotation at 4 °C. Subsequently, beads were washed in lysis buffer followed by isolation of RNA using Trizol and chloroform. cDNA was synthesized and subjected to qRT-PCR for the determination of binding of circNPHP1 and the miRNAs.

Fold enrichment was calculated for circNPHP1 and the miRNAs as follows: miRNA pulldown sample/control pulldown sample (X), miRNA input sample/control input sample (Y), fold enrichment = X/Y. A total of 10% of the cell extract was used as input for individual samples.

### Bromodeoxyuridine cell proliferation assay and annexin V apoptosis assay

After cell seeding (1 × 10^4^ cells/well) and cell transfection, the proliferation of HCMEC, HUVEC, AC16, or cardiac fibroblasts was determined by bromodeoxyuridine (BrdU) incorporation (for 6 hours) using the BrdU Cell Proliferation ELISA kit (Abcam) following manufacturer’s instructions. Cell death was measured using *the* Annexin V Apoptosis and Necrosis Assay RealTime-Glo kit (Promega) in complete medium following manufacturer’s instructions.

### Endothelial cord formation assay

After transfection with circNPHP1 siRNA, linear NPHP1 siRNA, or control siRNA, HUVECs (13 × 10^4^ per well) or HCMECs (15×10^4^ per well) were seeded on a 96-well plate coated with 50 μL Growth Factor Reduced Matrigel (Corning). After 8 hours, cells were fixed with 4% formaldehyde followed by permeabilization with Triton X-100 and staining with Phalloidin (Thermo Fisher Scientific). Images were captured at ×4 magnification using a Zeiss Axio Observer inverted microscope. Cord formation was analyzed using the angiogenesis analyzer plugin for the National Institutes of Health ImageJ software.

### Western blot analyses

Cells were harvested and lysed in ice-cold RIPA buffer (Sigma-Aldrich) containing 1 mmol/L orthovanadate, 1 mg/mL of protease inhibitor cocktail (Roche). Protein concentrations were determined using Bradford assay reagent (BIORAD). Equal amounts (15 μg) of proteins were fractionated in 4% to 20% SDS-polyacrylamide gel electrophoresis and transferred onto a polyvinylidene difluoride membrane using the wet transfer method. The membranes were blocked with 5% milk followed by incubation with primary antibodies overnight using the following antibodies: anti-VEGF-A (Abcam), anti-BCL2 (Cell Signaling Technology), anti-Lamin B1 (Cell Signaling Technology). The membrane was further probed with anti-Rabbit IgG-HRP (Santa Cruz Biotechnology). Immobilon Crescendo Western HRP substrate (Merck Millipore) was used to detect the chemiluminescence. Protein bands were visualized using the ChemiDoc MP Imaging System (BIORAD). Densitometry analysis of the gels was carried out using National Institutes of Health ImageJ software. The details of antibodies are listed in the resources table.

### Statistical analyses and data management

The analyses of the RNA-seq data sets are included in the “Bioinformatics analyses of RNA-seq data” and “Single-cell and single-nuclei RNA-seq data analysis” sections of the Methods.

For the remaining data, statistical analyses were performed with GraphPad Prism version 10.5 (GraphPad Software). Normal distribution of each experimental group was determined by Shapiro-Wilk test. Statistical significance between 2 groups was analyzed by unpaired Student's *t*-test. Comparisons among multiple groups were performed using 1-way analysis of variance or Kruskal-Wallis test with Dunnett's or Dunn's post hoc test for multiple pairwise comparisons, respectively. Analysis of variance and Kruskal-Wallis were indicated in the figure legends. Data are presented using actual data points with the mean ± SEM or median with 25th-75th percentiles (eg, boxplots). *P* values <0.05 were considered statistically significant.

In adherence to FAIR policy of data management,^46^ the data created and/or analyzed in the current study will be made available through online repositories (see data availability section).

## Results

### Differentially expressed circRNAs, miRNAs, and mRNAs in LV biopsy samples

**Figure 2:**
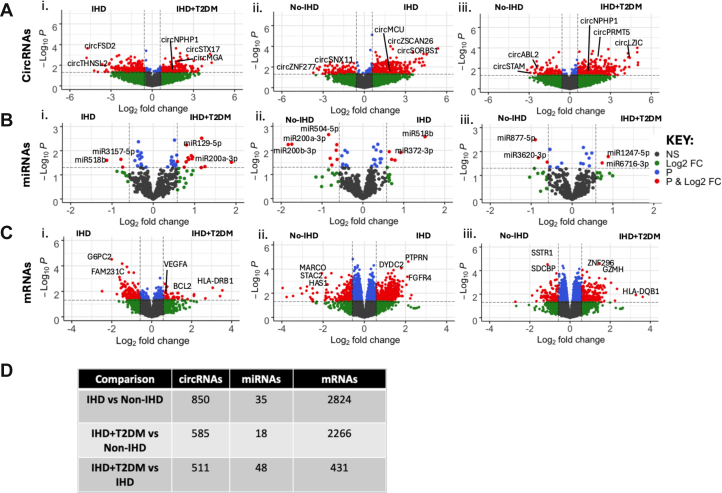
Differential Expression Analyses (A to C) CircRNAs, miRNAs and mRNAs were identified following the pipeline outlined in the Methods section: IHD (n = 12), IHD+T2DM (n = 11), non-IHD (n = 12). Differential expression analyses were performed on normalized and log-transformed counts using the R package *Limma.* Three comparisons were performed: 1) IHD+T2DM vs IHD; 2) IHD vs non-IHD; and 3) IHD+T2DM vs non-IHD. The corresponding differentially expressed circRNAs, miRNAs, and mRNAs were plotted in a volcano plot with log2 fold change (FC) on the x-axis and −log10 *P* value on the y-axis. CircRNAs, miRNAs, and mRNAs that passed the log2 FC threshold of ±0.58 and *P* value <0.05 are shown in red, those that just pass the FC threshold are in green, and those that only pass the *P* value threshold are in blue. (D) Number of significant differentially expressed circRNAs, miRNAs, and mRNAs in LV biopsies (log2 FC threshold of ±0.58 and *P* value <0.05). Abbreviations as in Figure 1.

Following the circRNA identification strategy described in methods and illustrated in Figure 1B, RNA-seq analyses were carried out. The number of detected circRNAs as well as common and uniquely expressed circRNAs among the 3 patient groups are shown in Figure 1C. We next performed differential expression analyses and identified the significantly DE circRNAs (Figure 2A), miRNAs (Figure 2B), and mRNAs (Figure 2C) (*P <* 0.05) in the following: 1) IHD vs IHD+T2DM; 2) no-IHD vs IHD; and 3) no-IHD vs IHD+T2DM. A table lists the number of differentially expressed circRNAs, miRNAs, and mRNAs in each comparison (Figure 2D). The files containing lists of significant DE circRNAs, miRNAs, and mRNAs relevant to each comparison between patient groups are provided as Supplemental Files 2A to 2C).

### CircRNAs are predicted to sponge miRNAs that affect mRNAs involved in EC- relevant pathways

**Figure 3:**
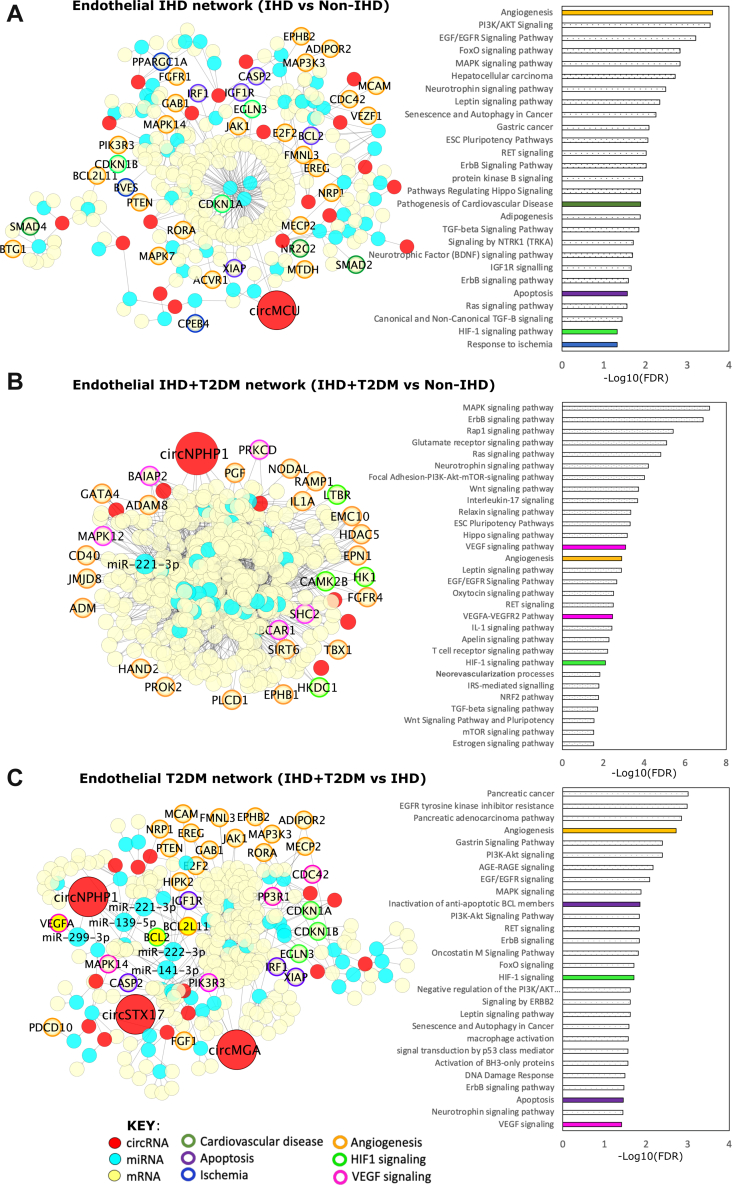
Predicted circRNA-miRNA-mRNA Networks in the Heart ECs (A to C) The endothelial circRNA-miRNA-mRNA networks were constructed in Cytoscape using predicted interactions among circRNAs, miRNAs, and mRNAs in IHD (± T2DM) (using in-house LV biopsies and heart ECs [GEO data]). All nodes (circRNAs, miRNA, and mRNAs) shown in the network are expressed in LV biopsies and ECs. The networks show circRNAs in red, miRNAs in cyan, and mRNAs in yellow. Following Gene Ontology (GO) and KEGG pathway analyses, all significant GO biological processes and KEGG pathways (passing threshold of FDR <0.05) were plotted as a bar graph with −log10(FDR) on the x-axis. Moreover, pathways relevant to EC biology are highlighted in the graph as solid colors. The corresponding genes (downstream targets of circRNAs and miRNAs) are outlined with the color of the relevant pathways. Genes that correspond to more than 1 pathway are outlined with multiple colors. (A) Network constructed from IHD vs non-IHD comparison. CircMCU emerged as the top significantly up-regulated circRNA (enlarged and labelled), which together with its miRNA and mRNA partners, mapped to EC-relevant pathways including apoptosis, angiogenesis, and HIF1 signaling. (B) Network constructed from IHD+T2DM vs non-IHD comparison. CircNPHP1 emerged as the top significantly up-regulated circRNA (enlarged and labelled) which together with its miRNA and mRNA partners mapped to EC-relevant pathways including VEGF signaling, angiogenesis and HIF1 signaling. miR-221-3p is also labelled as it was common with IHD+T2DM vs IHD network (shown in C) and relevant in EC pathways. (C) Network constructed from IHD+T2DM vs IHD comparison. CircNPHP1, circSTX17, and circMGA emerged as the top significantly up-regulated circRNAs (enlarged and labelled). Of particular interest was circNPHP1, which together with its miRNA (miR-221-3p, miR-222-3p, miR-141-3p, miR-139-5p, and miR-299-3p), and mRNA partners (VEGFA, BCL2, BLC2L11) mapped to EC-relevant pathways including VEGF signaling, apoptosis, angiogenesis, and HIF1 signaling. All enlarged nodes indicating circRNAs were selected for preliminary screening in ECs. Abbreviations as in Figure 1.

Figure 3 shows the Cytoscape representations of the interactions (“edges,” shown as connecting lines) among the circRNAs, miRNAs, and mRNAs (shown as points called “nodes”). The networks indicate putative regulatory relationships between circRNAs, miRNAs and mRNAs for the 3 different comparisons between patient groups. Additionally, the 3 networks that are presented in Figure 2 have also been filtered based on expression of the RNAs in ECs by using published data on ECs from the Gene Expression Omnibus (see the Methods for details). Figure 3 thus illustrates the following: IHD vs non-IHD (EC-enriched, 305 nodes and 359 edges) (Figure 3A, left), IHD + T2DM vs non-IHD (EC-enriched, 449 nodes and 1,463 edges) (Figure 3B, left), and IHD + T2DM vs IHD (EC-enriched, 303 nodes and 361 edges) (Figure 3C, left). Following the networks creation, we looked at significant biological processes and pathways that these RNAs are expected to contribute to. All processes and pathways that were enriched in the network with FDR <0.05 were considered significant (Figures 3A to 3C, right). Significant processes and pathways include VEGF signaling, angiogenesis, apoptosis, and HIF-1 signaling, which are of possible relevance for vascular biology and disease (highlighted in colors in Figure 3). Additionally, the miRNAs (miR-221-3p, miR-222-3p, miR-139-5p, miR-299-3p, and miR141-3p) and mRNAs (VEGFA, BCL2, and BCL2L11) that were subsequently validated are labelled and highlighted in the networks (Figure 3C, left).

### Screening and validation of circRNAs predicted to be commanding EC networks involved in EC survival regulation

**Figure 4:**
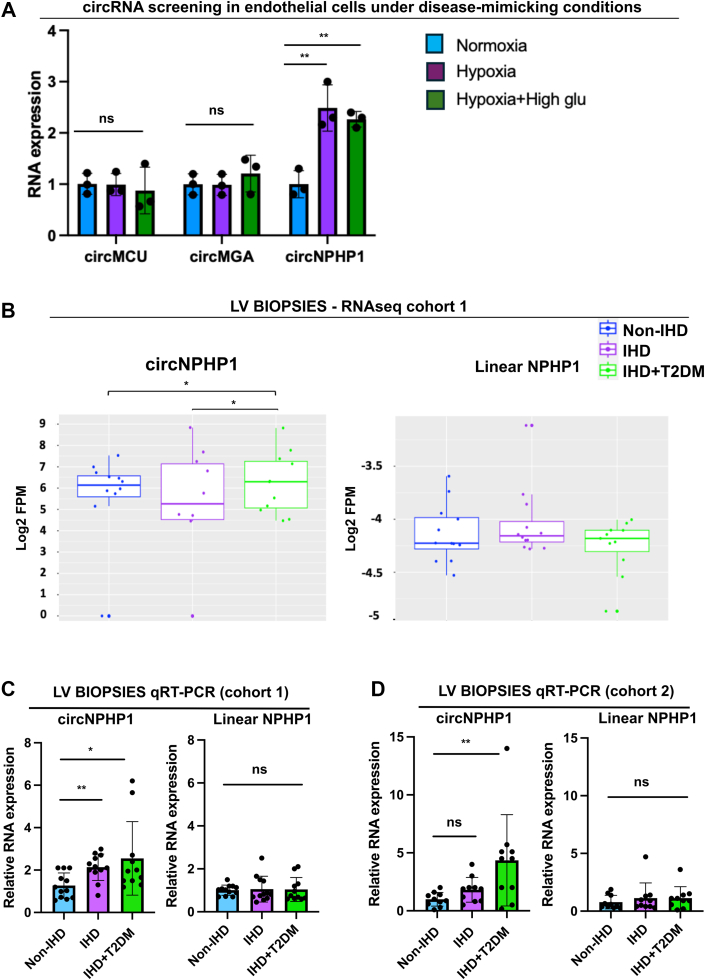
CircNPHP1 Expression in the Human LV and Cultured ECs Cardiac CircNPHP1 levels are higher in patients with IHD with/without associated T2DM and in cultured endothelial cells (human cardiac microvascular endothelial cells and human umbilical vein endothelial cells) exposed to disease-mimicking conditions, such as hypoxia and hypoxia, combined with increased D-glucose level (High glu). (A) Preliminary screening of circRNAs (identified from predicted networks) in endothelial cells: human cardiac microvascular endothelial cells were cultured in normal conditions, hypoxia (1% Oxygen), and hypoxia-high glucose (High Glu) (25 mmol/L D-glucose) conditions, respectively. After 48 hours, cells were harvested for quantitative real-time polymerase chain reaction (qRT-PCR) for the analysis and validation of indicated circRNAs. RNA expression is relative to normoxia. 18S is used as housekeeping gene; n = 3. Data are expressed as *mean ± SEM* and were assessed by 1-way analysis of variance with Dunnett's post hoc test. ∗*P <* 0.05, ∗∗*P <* 0.01, ∗∗∗*P <* 0.001, and ns = not significant. CircSTX17 was also tested but the levels were undetectable; hence, it is not included in the plot. CircNPHP1 emerges as an interesting candidate for further exploration and validation. (B) Comparison of expression levels of circNPHP1 and its linear counterpart in different patient groups—RNAseq (LV biopsies, cohort 1). Log-transformed normalized (FPM) counts of circNPHP1 and linear NPHP1 are plotted as a boxplot showing the median and IQR. Comparison between patient groups and calculation of *P* values was done using the R package Limma. CircNPHP1 is significantly up-regulated in IHD+T2DM compared with IHD and non-IHD (*P <* 0.05). Linear NPHP1 is expressed at very low levels and does not change between the different patient groups (IHD [n = 12], IHD+T2DM [n = 11], non-IHD [n = 12]). (C) qRT-PCR of circNPHP1 (left) and linear NPHP1 (right) of LV biopsies (cohort 1) from non-IHD (n = 12), IHD (n = 12), and IHD+T2DM (n = 11), and (D) of LV biopsies (cohort 2) from non-IHD (n = 10), IHD (n = 10), IHD+T2DM (n = 10) of patients. RNA expression is relative to non-IHD; 18S is used as housekeeping gene. Data are expressed as *mean ± SEM* and were assessed by Kruskal-Wallis test followed by Dunn's post hoc test. ∗*P <* 0.05, ∗∗*P <* 0.01, ∗∗∗*P <* 0.001, and ns = not significant. Abbreviations as in Figure 1.

The network predictions were used as a guide to perform experiments. From each network, we selected circRNAs involved in EC-relevant enriched pathways such as angiogenesis and VEGF signaling (large red-labelled nodes in Figures 3A to 3C, left). These included circNPHP1 (Figures 3B and 3C), circMCU (Figure 3A), circSTX17, and circMGA (Figure 3C). Because these circRNAs had EC expression as well as relevance in EC biology, we performed pilot expressional analyses in cultured HCMECs under disease-mimicking conditions and confirmed that circNPHP1 increased in response to hypoxia with/without HG vs control, whereas other circRNAs (circMGA, circMCU, and circSTX17) did not show changes between the groups (Figure 4A). CircNPHP1 expression is consistent with the results of bioinformatics analyses (Figures 2 and 3). Hence, it was selected for continuing the study. To validate the circNPHP1 and linear NPHP1 results from the initial LV RNA-seq analyses (Figure 4B), qRT-PCR analyses were independently conducted in ventricle samples from the ARCADIA first cohort (Figure 4C) and second cohort, specifically recruited for data validation by PCR (Figure 4D). In both cohorts, circNPHP1 was confirmed to increase in IHD with/without T2DM vs non-IHD control. Additionally, in the validation cohort-2, circNPHP1 expression followed an increased trend in IHD without T2DM vs non-IHD control.

**Figure 5:**
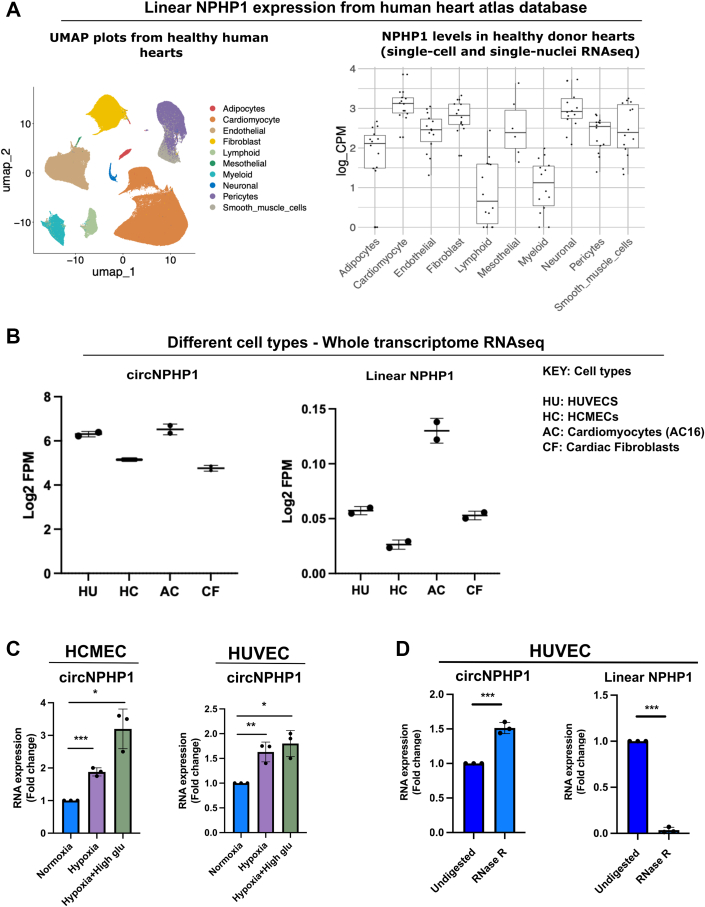
CircNPHP1 and Linear NPHP1 Expression in Different Cell Types (A) Single-cell and single-nuclei RNAseq data from healthy donor hearts (human heart atlas^42^) of NPHP1 gene as log2 transformed counts per million (CPM). Each dot indicates the data point as 1 donor (source: human heart atlas^42^). (B) RNAseq: A preliminary screen was carried out in different cells (sample size = 2 each) to determine absolute levels of circNPHP1 and linear NPHP1 in human umbilical vein endothelial cells (HUVECs or HU), human cardiac microvascular endothelial cells (HCMECs or HC), human AC16 cardiomyocytes (ACs), and cardiac fibroblasts (CFs). (C) HCMEC (left) and HUVEC cells (right) were cultured in either normal conditions, hypoxia (1% oxygen), or hypoxia combined with high glucose (25 Mm D-glucose). After 48 hours, cells were harvested for quantitative real-time polymerase chain reaction for the analysis of circNPHP1 (n = 3). Fold change in RNA expression is relative to normoxia; 18S is used as housekeeping gene. Data are expressed as *mean ± SEM* and were assessed by 1-way analysis of variance with Dunnett's post hoc test. ∗*P <* 0.05, ∗∗*P <* 0.01, ∗∗∗*P <* 0.001, and ns = not significant. (D) 2 μg of total RNA from HUVEC cells were digested with RNase R enzyme followed by quantitative real-time polymerase chain reaction analysis of circNPHP1 and linear NPHP1 (n = 3). Fold change in RNA expression is relative to undigested RNA; 18S is used as housekeeping gene. Data are expressed as *mean ± SEM* and were assessed by unpaired Student's *t*-test. ∗*P <* 0.05, ∗∗*P <* 0.01, ∗∗∗*P <* 0.001, and ns = not significant. Abbreviations as in Figure 1.

We next interrogated single-cell and single-nucleus RNA-seq data from the Human Heart Atlas^42^ (Figure 5A) to assess NPHP1 expression across cardiac cell types. Because standard scRNA-seq cannot detect circRNAs, we examined the linear isoform, assuming that cells expressing linear NPHP1 could also generate its circular counterpart. These analyses showed NPHP1 to be ubiquitously expressed across cardiac cell types. We next performed whole-transcriptome bulk RNA-seq of individual cardiac cells revealed higher circNPHP1 levels in endothelial cells and cardiomyocytes, with lower expression in cardiac fibroblasts (Figure 5B, left). In contrast, the linear isoform was expressed at very low levels in all cell types (Figure 5B, right). Normalized circRNA and mRNA counts for each cell type are provided in Supplemental File 4.

The regulation of circNPHP1 expression under hypoxia or combined hypoxia and HG conditions (compared with standard culture conditions) was further validated using independent batches of HCMECs and HUVECs (Figure 5C). The data obtained from ECs exposed to disease-mimicking environments demonstrated substantial increase in circNPHP1 expression, consistent with the qRT-PCR findings from ARCADIA patient samples.

To verify the circular nature and stability of circNPHP1, we performed an RNase R digestion assay, which selectively degrades linear RNAs without affecting circular forms. As anticipated, endogenous circNPHP1 exhibited resistance to RNase R treatment, whereas linear NPHP1 transcripts were markedly reduced (Figure 5D).

### CircNPHP1 binds to and sponges miR-221-3p in ECs

**Figure 6:**
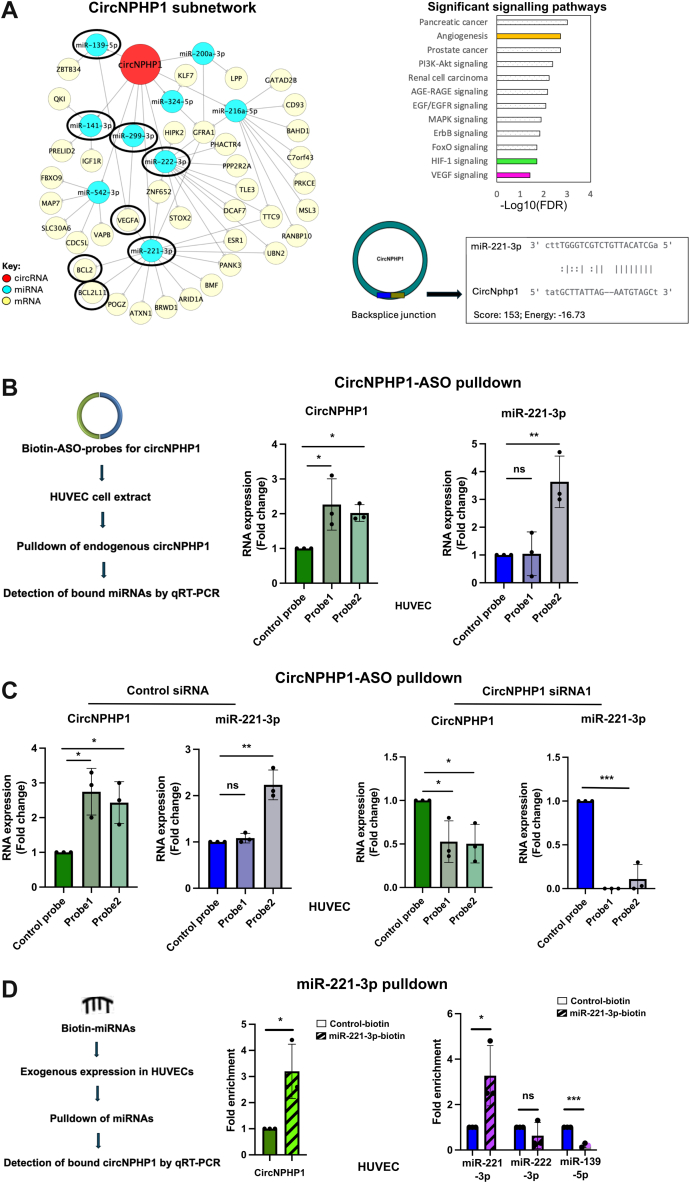
Identification and Validation of the CircNPHP1 Proangiogenic Interactome in Endothelial Cells CircNPHP1 is physically associated with the antiangiogenic miR-221-3p to putatively command a proangiogenic subnetwork. (A) CircNPHP1 subnetwork extracted from T2DM network (left). CircNPHP1 is connected to many miRNAs and mRNAs (circled in black) that mapped to EC-relevant pathways (VEGF signaling and angiogenesis; right, top). In particular, miR-221-3p bound exclusively to circNPHP1 via its back-splice junction sequence (right, bottom). The arc consisting of circNPHP1, its sponged miRNA partners (miR-221-3p, miR-222-3p, miR-299-3p, miR-141-3p, and miR-139-5p) and downstream targets (VEGFA, BCL2, BCL2L11) were selected for further validation. (B) CircNPHP1 ASO (anti-sense oligonucleotide) pulldown assay: HUVEC cell extracts were incubated with biotinylated ASO targeting the back-splice junction of circNPHP1 (probes 1 and 2) or control probe (nontargeting sequence) (see Supplemental Figure 1A for the details). Subsequently, the pulldown of endogenous circNPHP1 was carried out using streptavidin beads (schematic representation; see details in Methods section). The precipitated RNA was subjected to quantitative real-time polymerase chain reaction (qRT-PCR) for the analysis of circNPHP1 (left) and miR-221-3p (right). Fold enrichment was calculated against the control probe pulldown. 18S and U6 were used as reference genes to normalize for circNPHP1 and miR-221-3p respectively (n = 3). (C) HUVECs were transfected with 30 nmol/L of circNPHP1 siRNA (siRNA1) or control siRNA. 48 hours post-transfection, cells were harvested and incubated with biotinylated ASO control probe, probe 1, and probe 2, respectively, for the pulldown of circNPHP1. Subsequently, the precipitated RNA was subjected to qRT-PCR for the analysis of circNPHP1, linear NPHP1, and miR-221-3p. Fold enrichment was calculated relative to the control probe pulldown. 18S and U6 were used as reference genes to normalize for circNPHP1, linear NPHP1, and miR-221-3p, respectively (n = 3). Data (B and C) are expressed as *mean ± SEM* and were assessed by 1-way analysis of variance with Dunnett's post hoc test. ∗*P <* 0.05, ∗∗*P <* 0.01, ∗∗∗*P <* 0.001, and ns = not significant. (D) miRNA pulldown assay: HUVECs were transfected with 50 nmol/L of biotinylated miRNAs (miR-221-3p, miR-222-3p, miR-139-5p, respectively) or control biotinylated miRNA (scrambled nontargeting sequence). 48 hours post-transfection, cells were harvested and incubated with streptavidin beads for pulldown of the miRNAs (schematic representation; see details in Methods section). Subsequently, the precipitated RNA was subjected to qRT-PCR for the analysis of circNPHP1 (left) and the miRNAs (right). Fold enrichment (n = 3) was calculated in the pulldown samples as follows: miRNA pulldown/ control pulldown (X); miRNA input/ control input (Y), fold enrichment = X/Y. 10% of the cell extract was used as input for individual samples. Data are expressed as *mean ± SEM* and were assessed by unpaired Student's *t*-test between control and miRNA pulldown group. ∗*P <* 0.05, ∗∗*P <* 0.01, ∗∗∗*P <* 0.001, and ns = not significant. Abbreviations as in Figure 1.

CircNPHP1 as well as its partner miRNAs and mRNAs in the T2DM network mapped to a number of EC-relevant pathways including angiogenesis, VEGF signaling and HIF1 signaling (Figure 3), which indicates that circNPHP1 might be involved in various pathways through a combination of miRNA and mRNA partners. We, therefore, extracted the subnetwork of circNPHP1 from T2DM network. In total, 9 miRNAs were predicted to be sponged by circNPHP1 and 38 mRNAs to be potential target genes of the 9 miRNAs (Figure 6A, left, and pathway analyses, right). Using sequence analyses to investigate the miRNA interactome of circNPHP1, we identified miR-221-3p to be uniquely associated with circNPHP1, in contrast to other circular isoforms of NPHP1 and the linear NPHP1 transcript. Notably, the binding site for miR-221-3p is located within the back-splice junction of circNPHP1 (Figure 6A, right, bottom).

Although miR-221-3p also interacts with circNPHP1 within the IHD+T2DM network, its downstream mRNA targets differ from those identified in the T2DM network, thereby implicating distinct biological pathways.

To validate the interaction of miRNAs with circNPHP1 (circled in black; Figure 6A, left), pulldown of circNPHP1 was carried out in the HUVECs by using antisense oligo probes (probes 1 and 2) specifically designed against the sequence of its back-splice junction (Supplemental Figure 1A). The pulldown of circNPHP1 and the enrichment of the miRNAs was validated using qRT-PCR. The results showed effective enrichment of circNPHP1 pulldown by both the probes (Figure 6B). Following the circNPHP1 pulldown, we screened for the association of proangiogenic miRNAs, including miR-221-3p, miR-222-3p, miR-141-3p, miR-299-3p, and miR-139-5p. Among these, only miR-221-3p was significantly enriched, indicating a specific interaction with circNPHP1 (Figure 6B, Supplemental Figure 1B). This finding further supports the predicted exclusive binding of miR-221-3p to the back-splice junction of circNPHP1. Of note, the miR-221-3p enrichment was effective in the case of probe 2 but not in probe1. A possible explanation could be steric hindrance in binding of miR-221-3p caused by other factors bound to probe 1 because of larger size. Importantly, knocking down the circNPHP1 in HUVECs before pulldown for the circRNA abolished the enrichment of miR-221-3p, strengthening the evidence of binding between miR-221-3p and circNPHP1 (Figure 6C). The linear NPHP1 was only detectable in the whole-cell extracts and was undetectable in the corresponding circNPHP1-enriched conditions, indicating specific pulldown of the circular RNA (Supplemental Figure 1C).

Using a reverse approach, we performed pulldown assays targeting miRNAs implicated in angiogenesis and EC proliferation (ie, miR-221-3p,^47^^,^^48^ miR-222-3p,^48^ and miR-139-3p^49^). The enrichment of circNPHP1 in the pulldown complexes was assessed via qRT-PCR. Our results demonstrate that among the 3 miRNAs, circNPHP1 was enriched exclusively in the miR-221-3p pulldown (Figure 6D, Supplemental Figure 1D), further corroborating the specific interaction between circNPHP1 and miR-221-3p. Collectively, these findings identify miR-221-3p as a key binding partner of circNPHP1 in ECs.

To investigate if the putative circNPHP1/miR-221-axis was relevant to other cardiac cell types, we first measured the expression of both RNA molecules by qRT-PCR in human AC16 cardiomyocytes and human cardiac fibroblasts, before proceeding with the circNPHP1 pulldown experiment. Expression of circNPHP1 and miR-221-3p in AC16 was well-detected and comparable to HUVECs (mean count: 30 for circNPHP1, 19 for mir-221-3p). Although miR-221-3p exhibited good expression (mean count: 25) in the cardiac fibroblasts, the expression of circNPHP1 as well as the linear form was poor (mean count: 35) (Supplemental Figure 2A). In AC16, probe 2 induced effective pulldown of circNPHP1, which was associated with enrichment of miR-221-3p (Supplemental Figure 2B). Because of poor expression of circNPHP1 in cardiac fibroblasts, the pulldown of circNPHP1 by either probe was undetectable. Despite the ineffective pulldown of circNPHP1, we still found enrichment of miR-221-3p in cardiac fibroblasts by both the probes (Supplemental Figure 2C). This could be possibly caused by significant binding of circNPHP1 to miR-221-3p which caused enrichment in miR-221-3p even with slight pulldown of circNPHP1.

### CircNPHP1 promotes proliferation and capillary-like cord formation capacity of cultured ECs

The functional relevance of the circNPHP1/miR-221 subnetwork for the human endothelium was confirmed in HUVECs and HCMECs transfected with circNPHP1 siRNA or control siRNA and submitted to EC biology tests performed under normal, hypoxia, and hypoxia + HG conditions. Knockdown of circNPHP1 specifically down-regulated the circular NPHP1 form whereas linear NPHP1 remained unchanged, validating the specificity of the siRNA for the circular form of NPHP1. Additionally, the expression of linear NPHP1 remained unchanged under disease-mimicking culture conditions, further supporting the qRT-PCR findings from ARCADIA patient samples (Supplemental Figure 3A).

**Figure 7:**
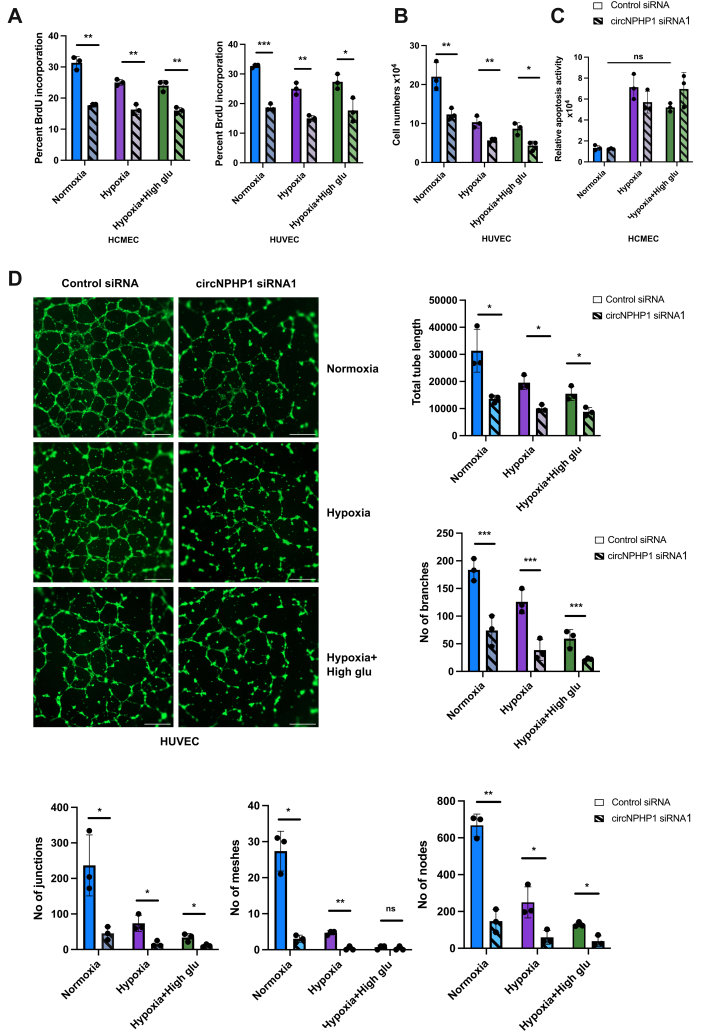
Impact of Endogenous CircNPHP1 on Endothelial Cell Proliferation, Apoptosis, and Cord Formation Endogenous CircNPHP1 promotes the proliferative and networking capacities of HUVECs and HCMECs. (A) HCMECs (left) and HUVECs (right) were transfected with 30 nmol/L of circNPHP1 siRNA or control siRNA, and cultured in normal conditions, hypoxia (1% oxygen), and hypoxia-high glucose (25 mmol/L D-glucose) conditions, respectively. 48 hours post-transfection, cells were harvested for proliferation assay (BrDU incorporation, 6 hours) (n = 3), (B) HCMEC: cells were counted, and cell numbers were determined using hemocytometer (n = 3) and (C) HCMEC: apoptosis assay (Annexin V assay) (n = 3). (D) HUVECs were transfected with 30 nmol/L of circNPHP1 siRNA or control siRNA, and cultured in normal conditions, hypoxia (1% oxygen), or hypoxia combined with high-glucose (25 mmol/L D-glucose) conditions. 48 hours post-transfection, the cells were seeded on a 96-well plate containing Growth Factor Reduced Matrigel and cultured in similar conditions for 8 hours to determine their capacity to network in a cord formation assay, which is a classical angiogenesis assay. Representative images of the cord formation (stained with Phalloidin, scale bar: 500 μmol/L). Histograms depicting the total tube length, number of branches, number of junctions, number of meshes and number of nodes quantified from the angiogenesis assay (n = 3). All of the data are expressed as *mean ± SEM*; results in (A to D) were by unpaired Student's *t*-test between control siRNA and circNPHP1 siRNA among the 3 groups, respectively. ∗*P <* 0.05, ∗∗*P <* 0.01, ∗∗∗*P <* 0.001, and ns = not significant. Abbreviations as in Figures 1 and 5.

Knockdown of circNPHP1 reduced EC proliferation, as evidenced by decreased BrdU incorporation (Figure 7A), along with a decline in total cell numbers across all experimental conditions (Figure 7B). By contrast, apoptotic death of the cells remained unaffected upon circNPHP1 knockdown in HCMECs (Figure 7C). Upon circNPHP1 knockdown in the HUVECs, cord formation assay on Matrigel showed prominent reduction of angiogenesis under standard culture conditions as well as in hypoxia and hypoxia+HG conditions (Figure 7D). The total tube length and the number of branches, meshes, nodes, and junctions exhibited significant reduction in the HUVECs with circNPHP1 knockdown (Figure 7D). Similar effect was observed in HCMECs (Supplemental Figure 3B). Taken together, our findings support the hypothesis that the circNPHP1/miR-221-3p axis plays a pivotal role in promoting EC proliferation and angiogenesis, forming a distinct proangiogenic subnetwork.

### Linear NPHP1 promotes proliferation and angiogenesis of cultured ECs

**Figure 8:**
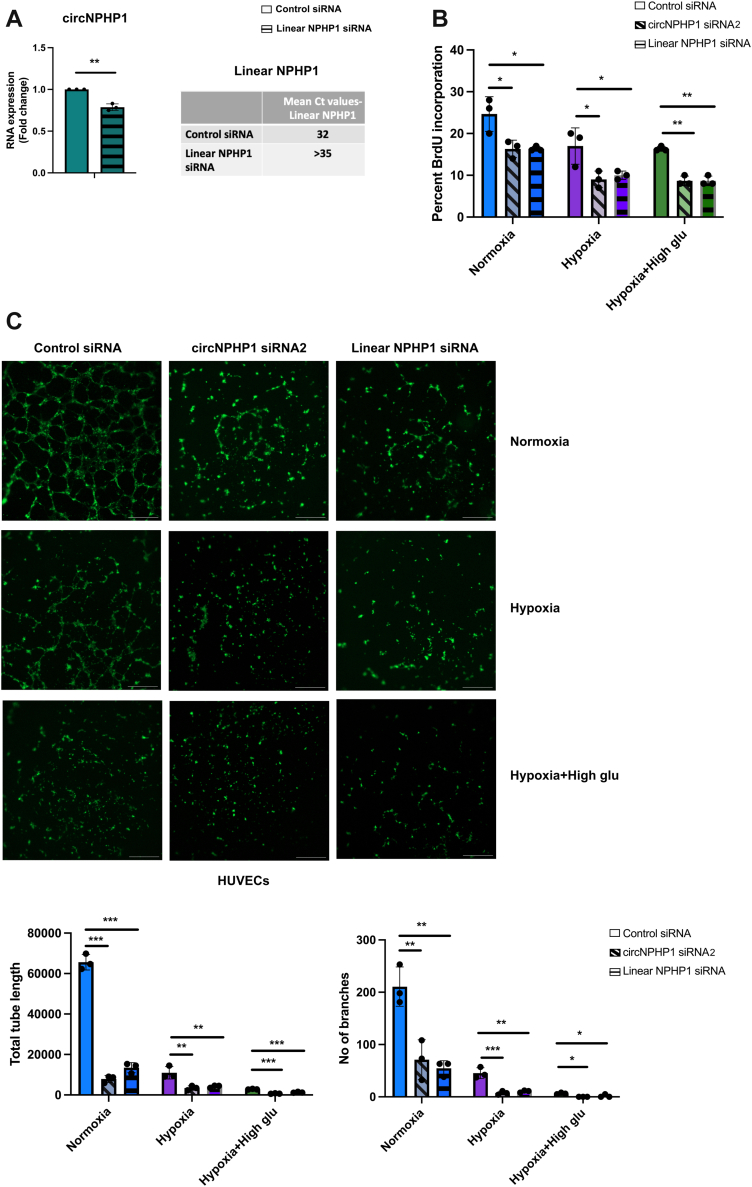
Linear NPHP1 Is Involved in Proliferation and Angiogenesis in the ECs (A) HUVECs were transfected with 30 nmol/L of linear NPHP1 siRNA or control siRNA. 48 hours post-transfection, cells were harvested for quantitative real-time polymerase chain reaction for the analysis of circNPHP1 and linear NPHP1 expression (n = 3). Fold change in mRNA expression is relative to control siRNA; 18S is used as housekeeping gene. Data are expressed as *mean ± SEM* and were assessed by unpaired Student's *t*-test. ∗*P <* 0.05, ∗∗*P <* 0.01, ∗∗∗*P <* 0.001, and ns = not significant. The table shows the average Ct values of linear NPHP1. Ct values >35 were considered undetectable. (B) HUVECs were transfected with 30 nmol/L of circNPHP1 siRNA2, linear NPHP1 siRNA, or control siRNA, and cultured in normal conditions, hypoxia (1% oxygen), and hypoxia-high glucose (25 mmol/L D-glucose) conditions, respectively. 48 hours post-transfection, cells were harvested for proliferation assay (BrDU incorporation, 6 hours) (n = 3) and (C) 48 hours post-transfection, cells were seeded on a 96-well plate containing Growth Factor Reduced Matrigel for 8 hours to determine cord formation. Representative images of the cord formation (stained with Phalloidin, scale bar: 500 μmol/L) (left). Histograms depicting the total tube length and number of branches quantified from the angiogenesis assay (right) (n = 3). Data are expressed as *mean ± SEM* and were assessed in (B and C) by 1-way analysis of variance with Dunnett's post hoc test between control siRNA, circNPHP1 siRNA2, and linear NPHP1 among the 3 groups, respectively. ∗*P <* 0.05, ∗∗*P <* 0.01, ∗∗∗*P <* 0.001, and ns = not significant. Abbreviations as in Figures 1 and 5.

We have also investigated the contributions of the linear NPHP1 in regulating cell proliferation and angiogenesis by silencing the linear NPHP1 using its specific siRNA. Knockdown of the linear NPHP1 markedly reduced its expression while circNPHP1 levels exhibited slight reduction (Figure 8A). This result possibly indicates independent processing of the linear and the circular forms of NPHP1 from the precursor mRNA.

To further validate the effect on cell proliferation and angiogenesis, we used a second siRNA for circNPHP1 (circNPHP1 siRNA2). Again, knockdown of circNPHP1 specifically down-regulated the circular NPHP1 form leaving the linear NPHP1 unchanged, validating the specificity of the second siRNA for circNPHP1 (Supplemental Figure 3C). In HUVECs, the knockdown of circNPHP1 by the second siRNA again showed marked reduction of the cell proliferation as measured by BrdU incorporation and prominent reduction of angiogenesis determined by cord formation assay across all the conditions (Figures 8B and 8C). Knockdown of the linear NPHP1 showed significant reduction of cell proliferation and angiogenesis as well. The total tube length and the number of branches exhibited prominent reduction with circNPHP1 knockdown by second siRNA as well as knockdown of linear NPHP1 (Figure 8C).

Altogether, our results strongly indicate that circNPHP1 is involved in promoting EC proliferation and angiogenesis. Our results also demonstrate significant contribution of linear NPHP1 in regulating EC proliferation and angiogenesis. It likely operates through independent mechanisms to that of the circNPHP1/miR-221 axis as the linear NPHP1 does not regulate circNPHP1 expression and remains unchanged in disease-mimicking conditions.

We have also profiled circNPHP1 and linear NPHP1 expression in HUVECs cultured only in HG conditions (mimicking diabetes without IHD). Both of the forms do not exhibit any significant changes between normal and high glucose conditions (Supplemental Figure 3D).

We have also checked the role of circNPHP1 in cellular functions of cardiac fibroblasts and AC16-cardiomyocytes. Interestingly, the proliferation of cardiac fibroblasts significantly decreased upon silencing circNPHP1 (Supplemental Figure 4A), whereas the cellular apoptosis in cardiac fibroblasts and AC16 remained unchanged (Supplemental Figure 4B). This suggests that the circNPHP1/miR-221-3p axis is operational in different cell types and could mediate different responses, which will need to be addressed in future studies.

### CircNPHP1 levels influence the expression of miR-221-3p target genes BCL2 and VEGFA in ECs

**Figure 9:**
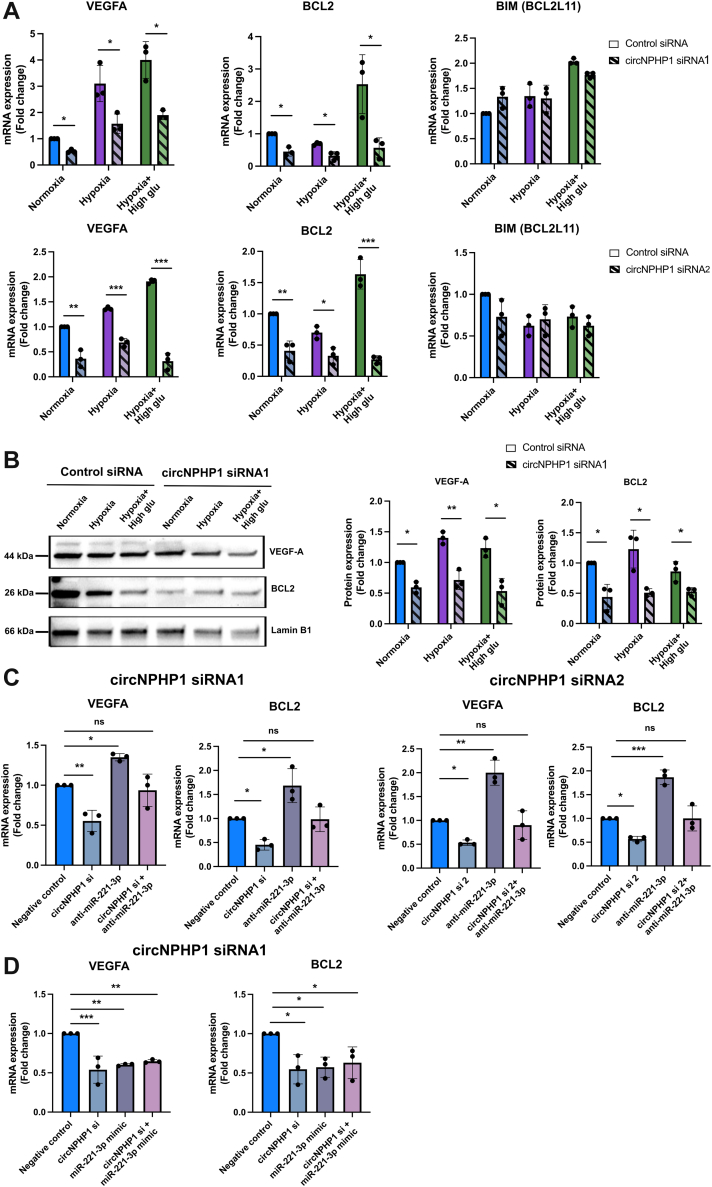
CircNPHP1 Regulation of miR-221-3p Target Genes’ Expression (A) HUVECs were transfected with 30 nmol/L of circNPHP1 siRNA (siRNA1: top or siRNA2: bottom) or control siRNA, and cultured in normal conditions, hypoxia (1% oxygen), and hypoxia-high glucose (25 mmol/L D-glucose) conditions, respectively. 48 hours post-transfection, cells were harvested for quantitative real-time polymerase chain reaction for the analysis of VEGFA, BCL2, and BIM (n = 3). Fold change in mRNA expression is relative to normoxia; 18S is used as housekeeping gene. (B) HUVECs were transfected with 30 nmol/L of circNPHP1 siRNA (siRNA1) or control siRNA and cultured as mentioned earlier. Cells were harvested for Western blot analysis of VEGF-A and BCL2 (n = 3). Images shown are representative (left). Histograms showing Western blot quantification are expressed as fold change against normoxia and normalized to Lamin B1 (loading control) (right). (C) HUVECs were transfected with 30nmol/L of circNPHP1 siRNA (siRNA1: left or siRNA2: right), anti-miR-221-3p, or in combination. Combination of control siRNA and anti-miRNA control is used as negative control. (D) 30 nmol/L of circNPHP1 siRNA (siRNA1), miR-221-3p mimic, respectively, or in combination. Combination of control siRNA and control mimic is used as negative control. 48 hours post-transfection, cells were harvested for quantitative real-time polymerase chain reaction for the analysis of VEGFA and BCL2 (n = 3). Fold change in mRNA expression is relative to negative control; 18S is used as housekeeping gene. All data are expressed as *mean ± SEM* and were assessed in (A and B) by unpaired Student's *t*-test between control siRNA and circNPHP1siRNA among the 3 groups, respectively, and in (C and D) by 1-way analysis of variance with Dunnett's post hoc test. ∗*P <* 0.05, ∗∗*P <* 0.01, ∗∗∗*P <* 0.001, and ns = not significant. Abbreviations as in Figures 1 and 5.

To confirm that circNPHP1 acts as a molecular sponge for miR-221-3p, thereby preventing it from repressing its target mRNAs, we focused on 3 validated miR-221-3p targets identified through our T2DM network analysis and supported by existing literature: BCL2, BCL2L11 (BIM), and VEGFA.^47^^,^^50^, ^51^, ^52^ These 3 genes corresponded to angiogenesis and cell survival pathways emerging from our pathway analyses in heart biopsies (FDR <0.05). We validated that the miR-221-3p mimic repressed VEGFA and BCL2 expression in HUVECs, whereas anti-miR-221-3p produced the expected opposite effects. By contrast, BIM was unaffected by the forced changes in miR-221-3p expression (Supplemental Figures 5A and 5B). Across all culture conditions, knockdown of circNPHP1 in HUVECs using 2 independent siRNAs (siRNA1 and siRNA2) resulted in decreased mRNA levels of VEGFA and BCL2 compared with cells transfected with control siRNA (Figure 9A). In contrast, BIM expression remained unaffected (Figure 9A). Therefore, BIM was excluded from further analyses. Western blots for VEGFA and BCL2 showed that protein expression was aligned with the mRNA expressional data (Figure 9B). To investigate if the changes in VEGFA and BCL2 expression induced by circNPHP1 silencing are mediated by the release of miR-221-3p repression, HUVECs were transfected with circNPHP1 siRNA (siRNA1 and siRNA2, respectively) or control siRNA in combination with an anti-miR221-3p. The inhibitory effect of circNPHP1 silencing on BCL2 and VEGFA was reduced when ECs were treated with anti-miR221-3p further confirming the joint operation of circNPHP1 and miR-221-3p in regulating VEGFA and BCL2 (Figure 9C). Silencing circNPHP1 in the presence of an miR221-3p mimic decreased VEGFA and BCL2 mRNA levels without showing a further reduction of mRNA expression in comparison to circRNA KD alone (Figure 9D).

## Discussion

Identifying the molecular defects that guide EC dysfunction in the human ischemic and diabetic heart could vastly improve the understanding of the pathology and consequently support the design and adoption of better preventive and curative treatment. RNA therapeutics are gaining momentum in experimental and clinical cardiology.^8^ Targeting the noncoding genome offers new perspectives,^19^ and an miRNA-targeting drug has reached phase 2 testing^9^ in cardiology. This antifibrotic drug represents the first successful attempt to reach to this advanced stage of translation of any noncoding RNAs. Despite initial promises,^53^^,^^54^ vascular therapies based on miRNA repression have not yet successfully proceeded through the sequential layers of in-human trialing. CircRNAs constitute an endogenous control mechanism capable to specifically regulate the post-translational activities of miRNAs. Targeting circRNAs to modulate miRNA activities represents a testable alternative to the direct miRNA targeting by lock nucleic acid or other oligonucleotide-based drugs. However, to the best of our knowledge, therapies targeting biological circRNAs remain untested in humans and represent a research field ripe for fundamental discoveries with the potential to ignite translational progress. Notably, the enhanced stability of synthetic circRNAs compared with linear mRNA forms is currently of considerable interest for the pharmaceutical industry,^55^ but to the best of our knowledge, has not yet been exploited to the therapeutical regulation of miRNAs.

The interactions between different RNA species are largely unexplored in clinically relevant models. We present here, for the first time, regulatory networks involving circRNAs, miRNAs, and mRNAs in the human heart affected by IHD leading to surgical revascularization. As part of this study, we have obtained an atlas of circRNAs, miRNAs, and mRNAs present in the human LV of patients with severe IHD associated or not associated with T2DM as well as in a control surgical group represented by non-IHD, nondiabetic patients undergoing repair of a mitral valve not compromised by a rheumatic etiology. The data have been obtained and analyzed as part of an observational prospective clinical study in cardiac surgery, expressively dedicated to noncoding RNA analyses in IHD and T2DM.

To guarantee the rigor of the work, the plan of analyses was designed and shared with the ethics committee ahead of accessing the RNA-seq data. Specifically, we identified circRNAs from whole transcriptome sequencing by putting together a pipeline using STAR and CIRCexplorer to detect back-spliced junctions. Several circRNA identification and annotation tools are currently available (reviewed by Panda^18^). For accurate identification of circRNAs from sequencing data, it is important to reduce false-positive rate of detection of back spliced junction reads. This can be achieved by considering detection of these junctions in several samples to gain more confidence or by applying a strict threshold on number of reads mapping to these junctions.^56^ Most circRNA identification tools depend on aligners such as Bowtie,^57^ BWA,^58^ and STAR.^2.7^ Several circRNA identification programs are available including Find_circ, CIRI,^59^ CIRCexplorer,^31^ CircTest,^60^ KNIFE,^61^ and so on. Among these, CIRCexplorer and KNIFE achieved the best sensitivity in detecting circRNAs,^62^ which is why we incorporated it in our detection pipeline. We also identified mRNAs and miRNAs from our whole transcriptome and small RNA-seq data, respectively. Following detection of the various RNA species and performing differential expression (DE) analysis, we used streamlined bioinformatics approaches to construct circRNA-miRNA-mRNA networks in IHD and IHD+T2DM with a particular focus on EC biology and processes mediating microvascular disease and repair (apoptosis, proliferation, angiogenesis). We also show that our networks are highly robust and that the number of nodes, network structure and parameters stayed intact whether filtering based on EC expression was performed before or after network creation (Supplemental Figure 6). Our study provides unique information on the putative interactions between these 3 RNA species (circRNA, miRNA, and mRNA) in IHD with/without T2DM and is a useful resource for developing testable hypothesis for future therapeutic studies. Our study highlights a novel proangiogenic subnetwork, possibly activated to counteract the ischemic damage by growing new microvessels, which is commanded by circNPHP1.

The role of circRNAs as sponges of miRNAs and their implication in heart and circulatory disease has been studied, mostly using animal models (revised in Barrett et al^15^ and Caporali et al^19^). A recent study predicted the circRNA-miRNA-mRNA interactions in the human postmyocardial heart at an advanced stage of dilation and failure.^24^ In this study, the LV samples were obtained from the noninfarcted remote myocardium, during surgical ventricular reconstruction procedure, or from hearts explants derived from transplantation procedures.^24^ The cell biology functional analyses were restricted to investigating circBPTF as regulator of cell death in HUVECs.^24^ Previous studies have demonstrated the associations between T2DM and circRNAs in diabetic cardiomyopathy in animal models. These include circRNA_000203 (MYO9A), circRNA_010567 (CDR1), and circHIPK3, which are increased in diabetic mice and promote myocardial fibrosis.^63^, ^64^, ^65^ We checked the levels of these circRNAs in our LV biopsy RNA-seq data sets and found circSLC8A1 and circHIPK3 to be increased in the LV of IHD with/out diabetes as compared with non-IHD patients whereas circMYO9A did not change and CDR1 was not detectable.

In the current study, circNPHP1 has been selected based on both expression in ECs and the predicted angiogenesis function of its commanded regulatory network encompassing downstream miRNA-mRNA partners. We next validated circNPHP1 to see whether it is up-regulated and promotes the proliferation of both HUVECs and HCMECs kept under normal and disease-mimicking conditions. CircNPHP1 did not influence apoptotic death. This suggests the prevalently reparative potential of circNPHP1 in the heart affected by CAD-induced angina, when the support of improved microcirculation could help boost the arrival of oxygens to the myocytes. Additional cardiac functions of this ubiquitously expressed circRNA are expected and deserve future investigation. To date, circNPHP1 has not been studied in the context of cardiovascular physiology and disease. CircNPHP1 is derived from exons 9 and 10 of the NPHP1 gene. Although other circular isoforms emerging from NPHP1of the circular RNA were identifiable in our RNA-seq data sets, their expression was scarce or represented in very few samples. The linear NPHP1 transcript was uniformly expressed in the healthy and diseased heart as evidenced from our RNAseq on heart LV biopsies and cells and confirmed by single nuclei RNA-seq analyses (human heart atlas), which indicated its expression to be ubiquitous, even if the binding partners and function might vary across different cell types. NPHP1 encodes for the protein Nephrocystin 1 that was initially discovered to be localized to the primary cilia and apical surface of kidney epithelial cells and interacts with molecules that take part in cell adhesion, signaling, and maintenance.^66^ NPHP1 has been associated with kidney disease (nephronophthisis) because of an autosomal recessive whole gene deletion resulting in abnormal structure and function of primary cilia.^67^ In nephronophthisis patients, cardiac defects like septal and aortic valve anomalies have also been reported.^68^ The direct involvement of NPHP1 in heart disease has not been reported to date, and information from GWAS Catalog (EMBL-EBI) indicates no involvement in IHD or T2DM. Worth noting is the fact that although the cardiac levels of NPHP1 do not vary either between IHD with/without T2DM and non-IHD patients (as from our bulk RNA-seq data) or between the human healthy and diabetic heart (as from single nuclei RNA-seq analyses), one of the circular isoforms of this gene (circNPHP1, studied here) does. Our bulk RNA-seq analysis revealed that circNPHP1 expression is significantly up-regulated in IHD associated with T2DM, compared with either IHD alone or healthy control subjects. Notably, circNPHP1 levels did not differ significantly between the IHD and control groups. In contrast, qRT-PCR analysis detected elevated circNPHP1 expression in both IHD groups (with and without T2DM) relative to control subjects. This discrepancy may reflect technical differences between the 2 platforms, particularly in data normalization procedures. However, qRT-PCR results from a second patient cohort aligned with the bulk RNA-seq findings, showing significant up-regulation of circNPHP1 only in the IHD+T2DM group. We speculate that the coexistence of IHD and T2DM may synergistically drive circNPHP1 up-regulation. Moreover, T2DM may exacerbate microvascular dysfunction, thereby amplifying myocardial ischemia and contributing to the observed expression pattern. Expanding the sample size in future studies could enhance statistical power and provide greater clarity regarding the observed expression patterns. The LV qRT-PCR findings align with data from ECs cultured under disease-mimicking conditions, including hypoxia and combined hypoxia with high glucose. Notably, hypoxia alone consistently elevated circNPHP1 expression in cultured EC, a pattern not fully mirrored in the LV qRT-PCR results. This discrepancy highlights the limitations of current in vitro models in faithfully recapitulating the complex pathophysiological environment of patients with chronic IHD and T2DM. Furthermore, high glucose alone did not alter circNPHP1 expression in cultured ECs, reinforcing the hypothesis that the co-occurrence of environmental stressors associated with IHD and T2DM may synergistically regulate circNPHP1 levels.

The linear form of NPHP1 remained unchanged between groups, as also indicated by single nuclei data in ECs resident in diabetic and nondiabetic heart donors. We also checked expression of circNPHP1 and linear NPHP1 in patient plasma samples from IHD with/without T2DM and non-IHD patients (Supplemental Table 3). The expression of circNPHP1 was not detectable across any of the groups, whereas linear NPHP1 exhibited very low expression. This indicates low release of both the forms from heart into plasma.

We found endogenous circNPHP1 to promote both EC proliferation and EC networking in capillary-like cords under hypoxia and HG (used to reflect the disease conditions in the dish). Other circRNAs were already reported to modulate endothelial function.^69^, ^70^, ^71^ However, in the context of human CAD leading to IHD, the presence, interactions, and functions of endothelial circRNAs remain largely unexplored. Additionally, we observed a significant contribution of linear NPHP1 in regulating EC proliferation and angiogenesis. This effect appears to be mediated through mechanisms independent of circNPHP1, as our data indicate that linear NPHP1 does not substantially regulate circNPHP1 expression, nor does circNPHP1 significantly influence the linear transcript. The slight reduction in circNPHP1 following inhibition of the linear form may reflect an indirect effect. Both linear and circular RNAs originate from the same pre-mRNA, and circularization (via back-splicing) often competes with canonical splicing in a context-dependent manner. Specific inhibition of the linear transcript may indirectly affect circRNA levels through cis-acting elements (ie, sequences within the linear form that influence circRNA production) or trans-acting factors, such as RNA-binding proteins modulated by the linear transcript that in turn regulate circRNA biogenesis. However, these regulatory processes are highly context-specific.^72^^,^^73^

Using our streamlined bioinformatics pipeline involving integration of different types of data including expression profiles of circRNA, miRNAs, mRNAs, published EC data, predictions on possible interactions between the three, and mapping of biological processes and pathways, we extracted a novel proangiogenic subnetwork commanded by circNPHP1 along with its predicted sponged miRNA partners (n = 9) and their downstream mRNA targets (n = 38). To confirm the interaction of the identified miRNAs with circNPHP1, pulldown of circNPHP1 was carried out in HUVECs by using antisense oligo probes (probes 1 and 2) designed against the sequence of its back-splice junction.^44^^,^^74^ Among the predicted miRNAs, we found only miR-221-3p to be effectively enriched. Although we predicted miR-222-3p as well, which is highly homologous to miR-221-3p and encoded in tandem from a gene cluster and often operating together,^75^ we did not observe enrichment in miR-222-3p. Although the pulldown of circNPHP1 with probe 1 was effective in the ECs, the enrichment of miR-221-3p with probe 1 was poor unlike the significant enrichment with probe 2. This could be explained considering that probe 1 has a larger size, which might bind other factors and can cause steric hindrance in binding of miR-221-3p. Additionally, circRNAs can possess certain conformational states, which might confer differential binding to various factors.^76^ Furthermore, in the miRNA pulldown assay targeting miR-222-3p, miR-221-3p was coprecipitated, whereas circNPHP1 was not enriched. This suggests potential competitive binding dynamics between circNPHP1 and miR-222-3p for interaction with miR-221-3p.

In the proangiogenic subnetwork of circNPHP1, miR-221-3p was found to interact with BCL2 and VEGFA, which are both involved in angiogenesis,^77^^,^^78^ and BIM (BCL2L11) that works together with BCL2 in a context-dependent manner. miR-221-3p expression was already reported to be elevated in the rat post-MI.^47^^,^^75^ The antiangiogenic role of miR-221-3p was previously demonstrated and explained with its repression of hypoxia inducible factor-1α (HIF-1α).^47^ Additionally, miR-221-3p targeting of angiopoietin-2 was reported to impair the proliferation and cord formation capacities of hypoxic HCMECs and to inhibit the formation of tip cells, which play a vanguard role in angiogenesis.^51^ In apparent contrast with these and our own data, miR-221-3p was proposed to promote proangiogenic and wound healing properties exerted by endothelial progenitor cell-derived exosome and to increase VEGF expression.^79^ VEGF-A has long been established to play a therapeutic angiogenic role in experimental models of MI.^80^, ^81^, ^82^ After several unsuccessful VEGF gene therapy clinical trials to induce regenerative angiogenesis in patients with cardiovascular disease,^83^ a VEGF-A protein therapy by a naked modified mRNA without lipid encapsulation (AZD8601), codeveloped by AstraZeneca and Moderna, successfully passed a randomized phase I study in otherwise healthy volunteers with T2DM (NCT02935712)^84^^,^^85^; next, it entered a randomized, double-blind, placebo-controlled multicentric safety study (EPICCURE) in IHD patients with moderately decreased LV function (ejection fraction 30%-50%) undergoing elective coronary artery bypass graft surgery^83^ (this population is similar to the ARCADIA IHD groups). AstraZeneca and Moderna discontinued the investment after announcing that the Phase 2a study met the primary endpoint of safety and tolerability and that exploratory outcomes indicated potential improvement in cardiac composition and function,^86^ even if the study was very limited in size (n = 11 patients in total for the 2 study groups in the study). Notwithstanding, these translational efforts have confirmed both the potential of promoting the local increase in soluble VEGF-A in the ischemic heart and the validity of nonviral delivery approaches. As discussed in the previous text, therapeutic circRNAs delivered via LNP could open new possibilities for improving the time-controlled regional increase of VEGF-A and other cardioprotective and regenerative proteins via microRNA sponging. Specifically to circNPHP1, BCL2 could be elevated concomitantly with VEGF-A. BCL2 also plays a crucial role in regulating the mitochondrial oxidative stress and angiogenesis in IHD^78^ and rat heart failure.^87^ Our results in ECs confirmed that endogenous circNPHP1 dictates VEGFA and BCL2 expression by sponging miR-221-3p. By contrast, BIM, which is largely proapoptotic,^88^^,^^89^ was unaffected upon modulation of circNPHP1.

We further investigated whether the circNPHP1/miR-221-3p axis is operational in other cell types. We also checked the binding of circNPHP1 to miR-221-3p in human AC16-cardiomyocyte and cardiac fibroblasts. The expression of linear and circNPHP1 in AC16 is comparable to that of the ECs, while cultured cardiac fibroblasts showed low expression of circNPHP1. Interestingly, in cardiac fibroblasts, circNPHP1 itself was barely detectable in the pulldown, whereas the associated miR-221-3p remained measurable. The reasons for this discrepancy remain unclear. Given that circNPHP1 contains only a single MRE for miR-221-3p, it is unlikely that each circNPHP1 molecule could bind multiple copies of the microRNA. Although a few exceptional circular RNAs—such as ciRS-7/CDR1as (for miR-7-5p)^90^ and circSRY (for miR-138-5p)^91^—are known to contain multiple MREs and act as potent miRNA sponges, this is not a generalizable feature of most circRNAs, including circNPHP1.

Functionally, knockdown of circNPHP1 reduced the proliferation of cardiac fibroblasts, while apoptosis remained unaffected. Although qRT-PCR showed low circNPHP1 expression, RNA-seq confirmed its presence in cardiac fibroblasts. It is plausible that even a modest reduction in circNPHP1 levels may impact critical downstream pathways essential for cell proliferation. Notably, our sequencing and PCR data also show that circNPHP1 is generally expressed at low levels in endothelial cells (ECs), yet it plays pivotal roles in EC function. These findings underscore the need for further investigation into the role of circNPHP1 in regulating cardiac fibroblast proliferation.

By contrast, circNPHP1 KD did not affect AC16 proliferation or apoptosis, suggesting that it might regulate other phenotypes. miR-221-3p could be coprecipitated with circNPHP1 in both AC16 and fibroblasts, suggesting that the circNPHP1/miR-221-3p axis is potentially operational in different cardiac cell types and the need for further investigation.

Modeling an mRNA regulatory circuit would ideally benefit from knowledge of the stoichiometric ratios between the interacting molecules. To achieve this level of absolute quantification, which corrects for artifacts introduced by PCR amplification, a method utilizing UMIs is required at the library preparation step. Although we did not employ UMIs in this study, our current approach includes postsequencing normalization^92^ to correct for technical variation between samples, such as differences in sequencing depth. However, the regulatory effect of circRNAs on miRNAs and their target mRNAs is not strictly dependent on exact molecule counts. As shown by Mukherji et al,^93^ miRNA-mediated repression exhibits threshold behavior, and robust repression often requires a substantial amount of miRNA relative to its targets. This relationship is nonlinear and context-dependent, particularly in complex cellular environments. Moreover, circRNAs can modulate miRNA activity through sponging mechanism, but the effectiveness of this regulation does not necessarily scale linearly with molecule abundance.^94^ Further studies employing site-directed mutagenesis of either circNPHP1 or miR-221-3p, as well as additional mechanistic experiments such as UV crosslinking or in vitro synthetic reconstitution of circRNA and miRNA could provide deeper insights into the nature of the interaction between circNPHP1 or miR-221-3p.

### Study limitations

The mechanistic part of this study has focused on EC and angiogenesis. Notwithstanding, the ARCADIA RNA-seqs allow for expanding this focus in multiple directions. Our data sets are available for further analyses by us and others and are expected to expand beyond the specific focus kept in this first study. In vivo assessment of the proangiogenic subnetwork with regard to cardiac functions and reparative angiogenesis in animal models remains to be explored. Furthermore, given the modest expression of circNPHP1 in cardiac fibroblasts, in-depth mechanistic insights and implications of interaction between circNPHP1 and miR-221-3p remain to be further investigated. Additionally, thorough investigation and functional implications of the circNPHP1/miR-221-3p in cardiomyocytes requires further investigation.

## Conclusions

Our study has uncovered novel, differentially regulated circRNA–miRNA–mRNA networks in IHD and IHD+T2DM with a particular focus on ECs. It specifically highlights the proangiogenic axis involving circNPHP1/miR-221-3p/VEGFA/BCL2 in IHD and IHD+T2DM implicating its potential significance for therapeutic angiogenesis and cardiac repair.

### Data Availability

The ARCADIA human left ventricle biopsy and cell bulk RNA-seq data sets are available via Sequence Read Archive (SRA) (https://www.ncbi.nlm.nih.gov/sra) database. BioProject accession number: PRJNA1336212.

Additional data sets created and/or analyzed during the current study are available upon request.

## Funding Support and Author Disclosures

This study was supported by the British Heart Foundation (RG/20/9/35101, RG/P/34397, and CH/15/1/31199 to Prof Emanueli) and the National Institute of Heart Research via the Imperial and Bristol Biomedical Research Centre (to Prof Angelini). The authors have reported that they have no relationships relevant to the contents of this paper to disclose.
