# Supplementary material for: Human Left Ventricle circRNA-miRNA-mRNA Network Analyses Reveal a Novel Proangiogenic Role for circNPHP1 Under Ischemic Conditions
Source: JACC Basic Transl Sci. 2026 Jan 20;11(2):101468. doi: 10.1016/j.jacbts.2025.101468 (PMC12859194; doi:10.1016/j.jacbts.2025.101468)
Supplement: Supplemental Material [file mmc1.pdf]

Supplemental File 5: Unedited gels

Complete unedited western blot: (Figure 9B)

A. (representative image used in figure 9B)

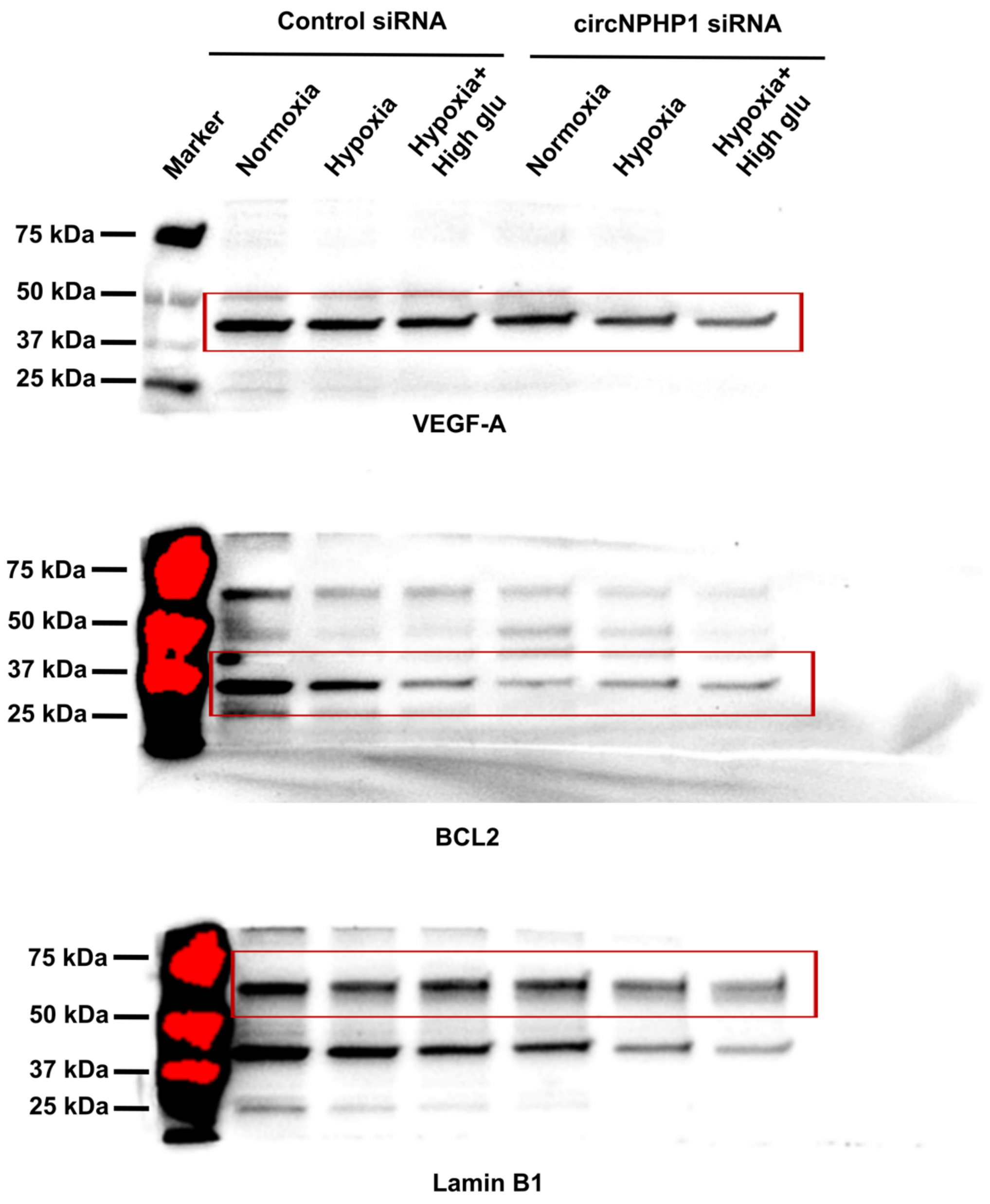

B.

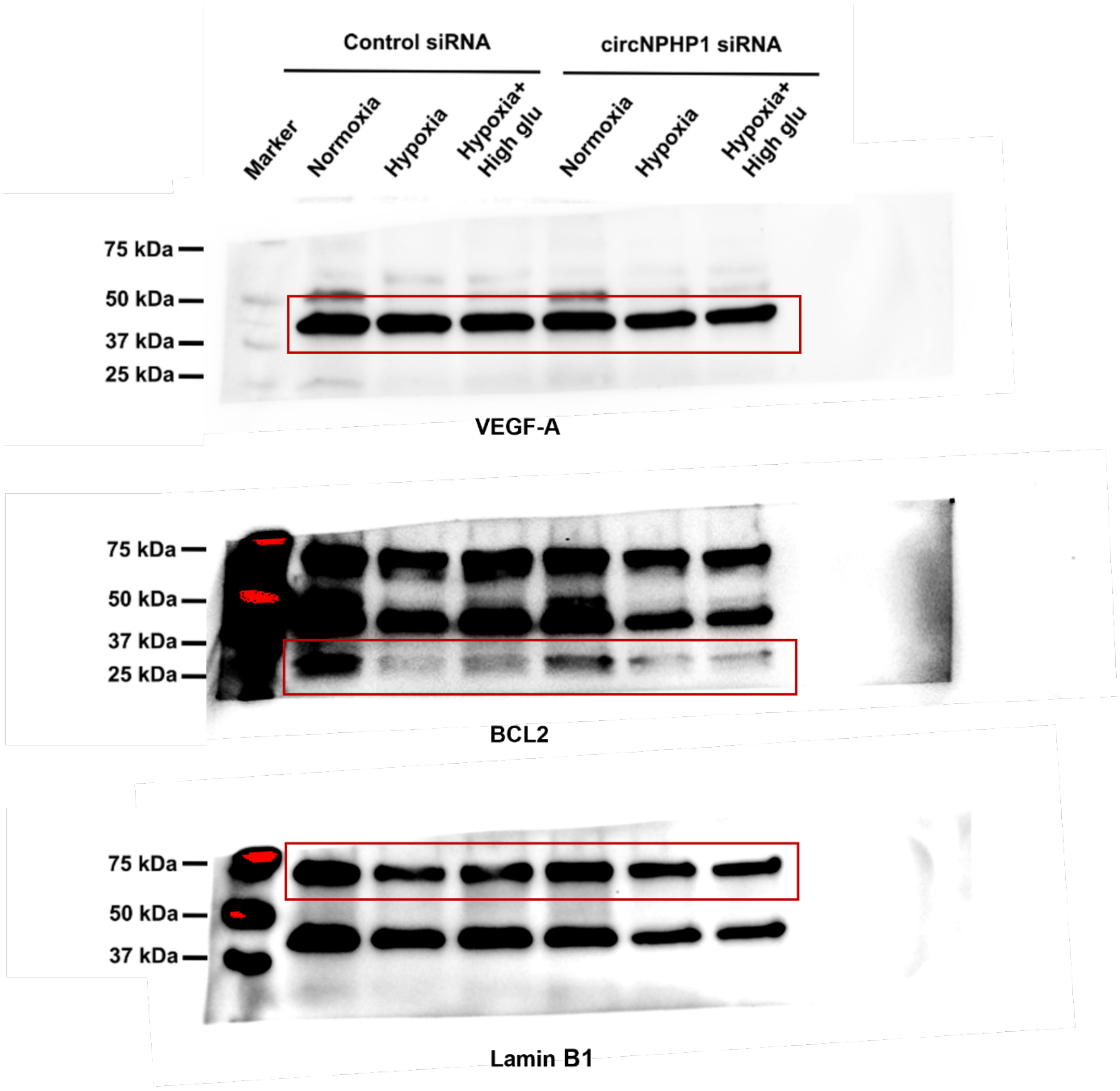
Control siRNA
circNPHP1 siRNA
Marker
Normoxia
Hypoxia
Hypoxia+ High glu
Normoxia
Hypoxia
Hypoxia+ High glu
75 kDa
50 kDa
37 kDa
25 kDa
VEGF-A
75 kDa
50 kDa
37 kDa
25 kDa
BCL2
75 kDa
50 kDa
37 kDa
Lamin B1

C.

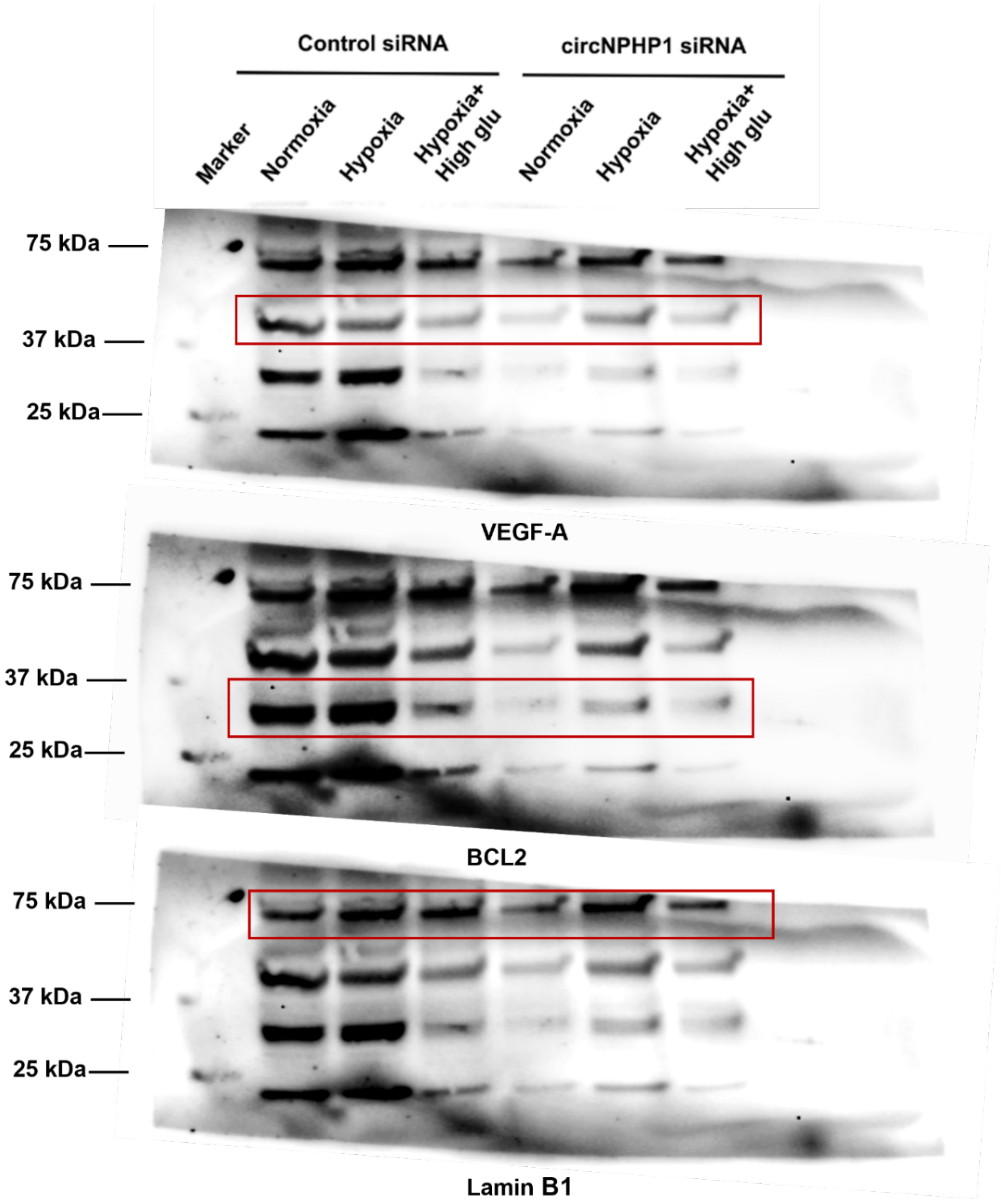

Unedited western blot: Unedited western blots with additional replicates used for **Figure 9B**

(A) Unedited blot of representative image used in **figure 9B (B) and (C**) are additional biological replicates. The red rectangle shows the respective protein lanes used in the representative image and additional quantifications.
