# Supplementary material for: Human Left Ventricle circRNA-miRNA-mRNA Network Analyses Reveal a Novel Proangiogenic Role for circNPHP1 Under Ischemic Conditions
Source: JACC Basic Transl Sci. 2026 Jan 20;11(2):101468. doi: 10.1016/j.jacbts.2025.101468 (PMC12859194; doi:10.1016/j.jacbts.2025.101468)
Supplement: Supplemental Appendix [file mmc2.docx]

**Supplemental Appendix**

**Contents Page numbers**

Title page 1

Supplementary Figure 1 2-3

Supplementary Figure 2 4-5

Supplementary Figure 3 6-7

Supplementary Figure 4 8

Supplementary Figure 5 9

Supplementary Figure 6 10-11

Supplementary Table 1 12

(Patient characteristics cohort 1)

Supplementary Table 2 13

(Patient characteristics cohort 2)

Supplementary Table 3 14

(Expression of CircNPHP1 and

linear NPHP1 in the plasma of patients)

**Supplementary Figure 1**


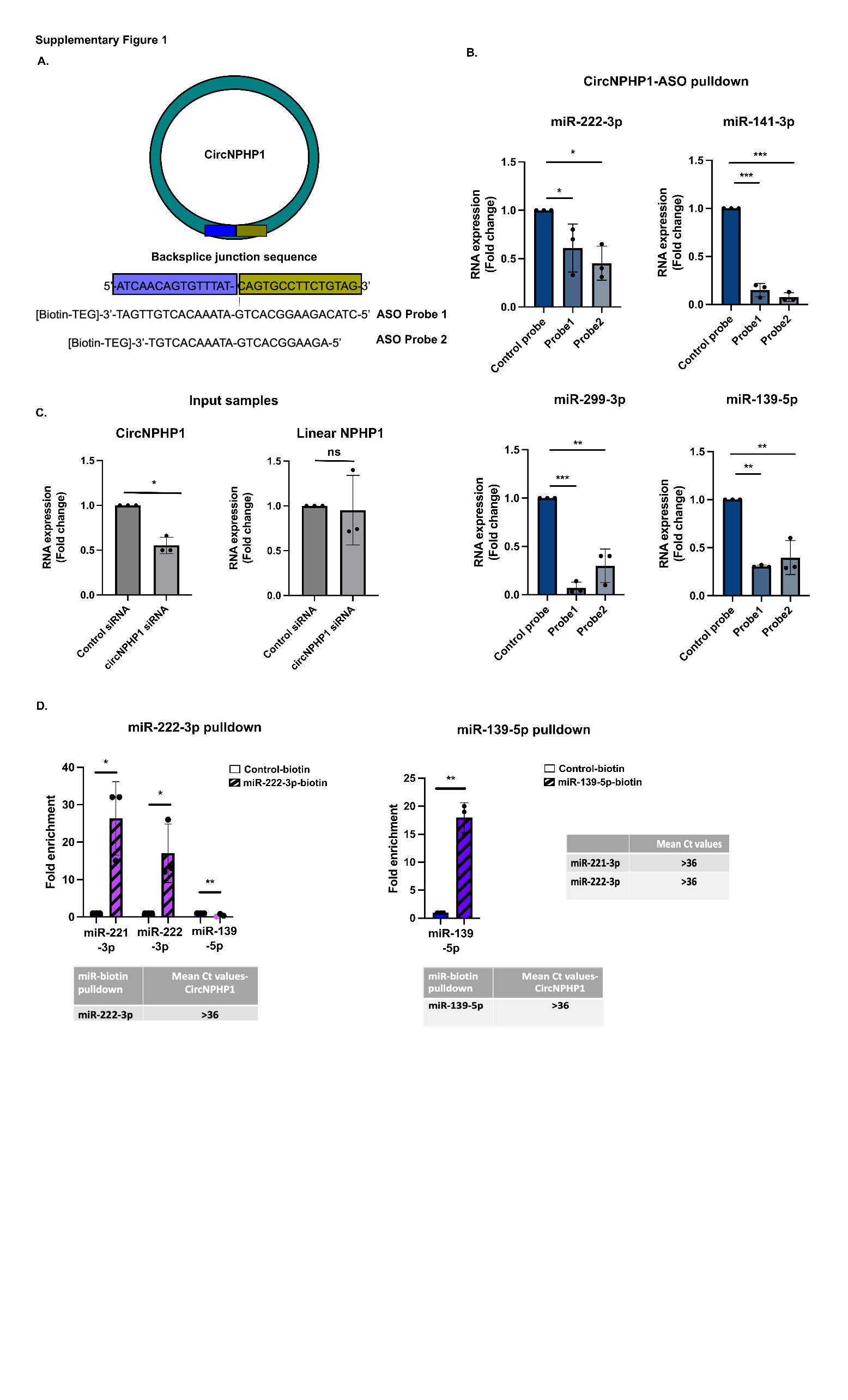


**Supplementary Figure 1: CircNPHP1 binds miR-221-3p and constitutes a pro-angiogenic sub-network**

(A) Schematic representation and sequence of the biotinylated probes used for pulldown of circNPHP1. Antisense oligo (ASO) probe 1 was designed by joining the last 15 nucleotides of circNPHP1 (green) to the first 15 nucleotides (blue) to make a 30-nucleotide sequence against the backsplice junction. The second probe (probe 2) consists of 22 nucleotide sequence against the backsplice junction, which is also the siRNA sequence for circNPHP1 used in this study.

(B) HUVECs extracts were incubated with biotinylated ASO targeting the back-splice junction of circNPHP1 (probe 1 and probe 2) or control probe (non-targeting sequence). Subsequently, the pulldown of circNPHP1 was carried out using streptavidin beads and the precipitated RNA was subjected to qRT-PCR for the analysis of miR-222-3p, miR-141-3p, miR-139-5p, miR-299-3p. Fold enrichment was calculated relative to the control probe pulldown. 18S and U6 were used as reference genes to normalize for circNPHP1, linear and the miRNAs respectively (N=3) (**Refer Figure 6B**).

(C) HUVECs were transfected with 30nM of circNPHP1 short interfering RNA (siRNA) or control siRNA. 48 hours post transfection, cells were harvested for circNPHP1 pulldown (**Refer figure 6C**) qRT-PCR for the analysis of circNPHP1 (left panel) and linear NPHP1 (right panel) (N=3) in the input samples. Fold change in RNA expression is relative to control siRNA; 18S is used as housekeeping gene.

(D) HUVECs were transfected with 50nM of biotinylated miRNAs (miR-222-3p and miR-139-5p respectively) or control biotinylated miRNA. 48 hours post transfection, cells were harvested and incubated with streptavidin beads for pulldown of the miRNAs. Subsequently, the precipitated RNA was subjected to qRT-PCR for the analysis of circNPHP1 and the miRNAs. Fold enrichment (N=3) was calculated in the pulldown samples as follows: miRNA pulldown/ control pulldown (X); miRNA input/ control input (Y), fold enrichment= X/Y. 10 percent of the cell extract was used as input for induvial samples. (**Refer Figure 6D**).

The tables (bottom panels) show the average cycle threshold (Ct) values of circNPHP1 in miR-222-3p and miR-139-5p pulldowns respectively. For miR-139-5p pulldown, Ct values of miR-221-3p and miR-222-3p are shown in the table (right panel). Ct values > 36 were considered undetectable.

Data are expressed as mean*±*SEM and were assessed in (B) by one-way ANOVA with Dunnett's post-hoc test and in (C and D) by unpaired Student *t* test. *P<0.05, **P<0.01, ***P<0.001, and ns = not significant.

**
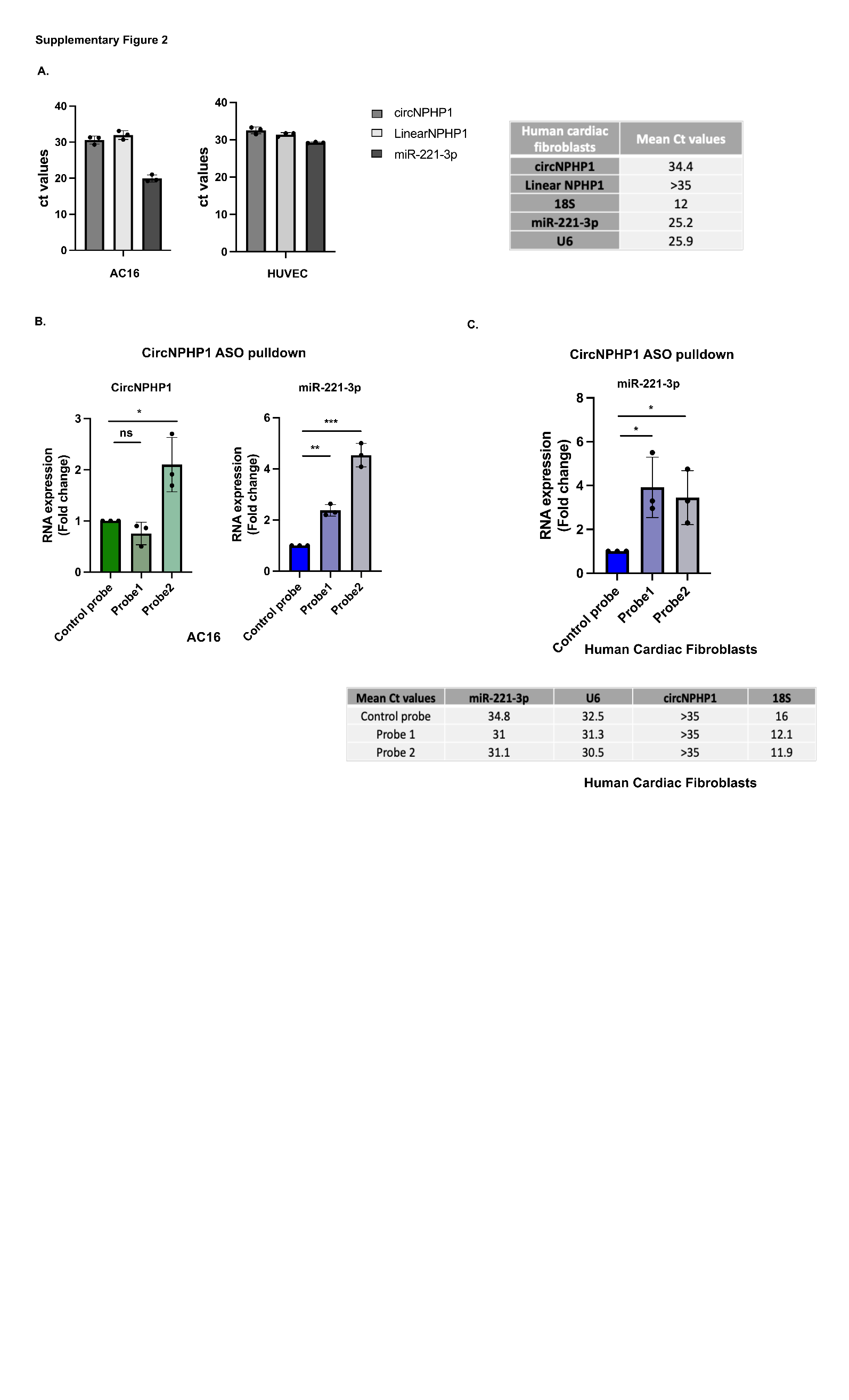
Supplementary Figure 2**

**Supplementary Figure 2: CircNPHP1 binds miR-221-3p in cardiomyocytes and cardiac fibroblasts**

(A) Histograms depicting the Ct values from qRT-PCR of circNPHP1, linear NPHP1 and miR-221-3p (N=3) in AC16, HUVEC and cardiac fibroblasts (table). Ct values > 35 were considered undetectable.

(B) AC16 (C) cardiac fibroblast extracts were incubated with biotinylated ASO targeting the back-splice junction of circNPHP1 (probe 1 and probe 2) or control probe (non-targeting sequence). Subsequently, the pulldown of circNPHP1 was carried out using streptavidin beads and the precipitated RNA was subjected to qRT-PCR for the analysis of circNPHP1 (left panel) and miR-221-3p (right panel) (N=3). Fold enrichment was calculated relative to the control probe pulldown. 18S and U6 were used as reference genes to normalize for circNPHP1 and miR-221-3p respectively (N=3).

The table in (C) shows the average Ct values from pulldown in cardiac fibroblasts. Ct values > 35 were considered undetectable. Biological replicates in AC16 and cardiac fibroblast are carried out on cells from different aliquots and passages of the same lot.

Data are expressed as mean*±*SEM and were assessed in (B and C) by one-way ANOVA with Dunnett's post-hoc test. *P<0.05, **P<0.01, ***P<0.001, and ns = not significant.

**
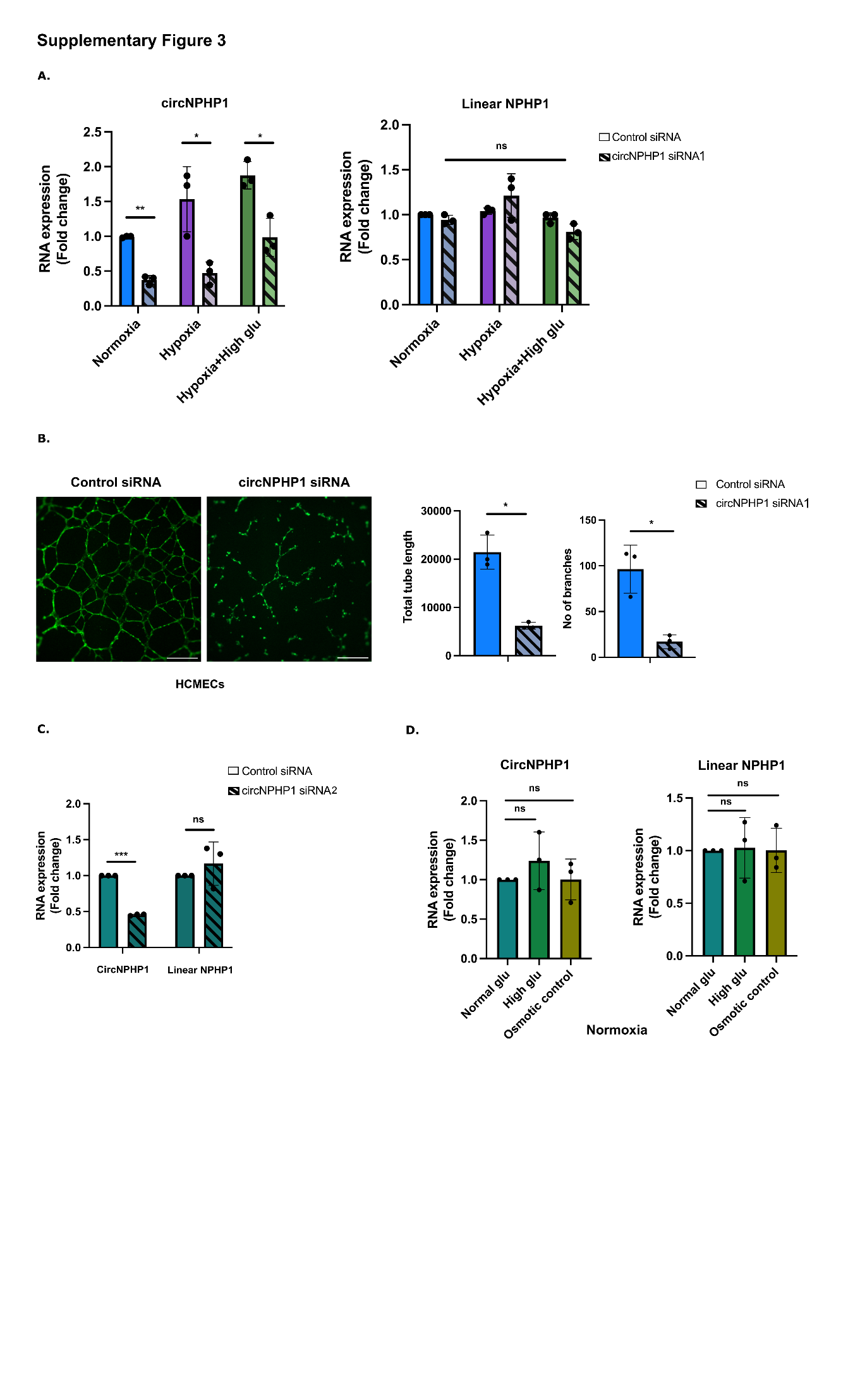
Supplementary Figure 3**

**Supplementary Figure 3: CircNPHP1 is involved in proliferation and angiogenesis in the ECs**

(A) HCMECs were transfected with 30nM of circNPHP1 short interfering RNA (siRNA) or control siRNA, and cultured in normal conditions, hypoxia (1% Oxygen) and hypoxia-high glucose (25mM D-glucose) conditions respectively. 48 hours post transfection, cells were harvested for qRT-PCR for the analysis of circNPHP1 (left panel) and linear NPHP1 (right panel) (N=3). Fold change in RNA expression is relative to control siRNA; 18S is used as housekeeping gene. Data are expressed as mean*±*SEM and were assessed by unpaired Student *t* test between control siRNA and circNPHP1 siRNA among the three groups respectively. *P<0.05, **P<0.01, ***P<0.001, and ns = not significant.

(B) HCMECs were transfected with 30nM of circNPHP1 siRNA or control siRNA. 48 hours post transfection, cells were seeded on a 96-well plate containing Growth Factor Reduced Matrigel for 8 hours to determine cord formation. Representative images of angiogenesis (stained with Phalloidin, scale bar: 500 mM) (left panel). Histograms depicting the total tube length and number of branches quantified from the angiogenesis assay (right panel) (N=3). Data are expressed as mean*±*SEM and were assessed by unpaired Student *t* test between control siRNA and circNPHP1 siRNA groups respectively. *P<0.05, **P<0.01, ***P<0.001, and ns = not significant.

(C) HUVECs were transfected with 30nM of circNPHP1 siRNA2 or control siRNA. 48 hours post transfection, cells were harvested for qRT-PCR for the analysis of circNPHP1 and linear NPHP1 expression (N=3). Fold change in mRNA expression is relative to control siRNA; 18S is used as housekeeping gene. Data are expressed as mean*±*SEM and were assessed by unpaired Student *t* test between control siRNA and circNPHP1 siRNA samples respectively. *P<0.05, **P<0.01, ***P<0.001, and ns = not significant.

(D) HUVECs were cultured in normal conditions, with medium containing normal glucose, high glucose (25mM D-glucose) and osmotic control (25mM I-glucose) conditions respectively. After 48 hours, cells were harvested for qRT-PCR for the analysis of circNPHP1 and linear NPHP1 expression (N=3). Fold change in mRNA expression is relative to normal glucose condition; 18S is used as housekeeping gene.

Data are expressed as mean*±*SEM and were assessed by one-way ANOVA with Dunnett's post-hoc test. *P<0.05, **P<0.01, ***P<0.001, and ns = not significant.

**
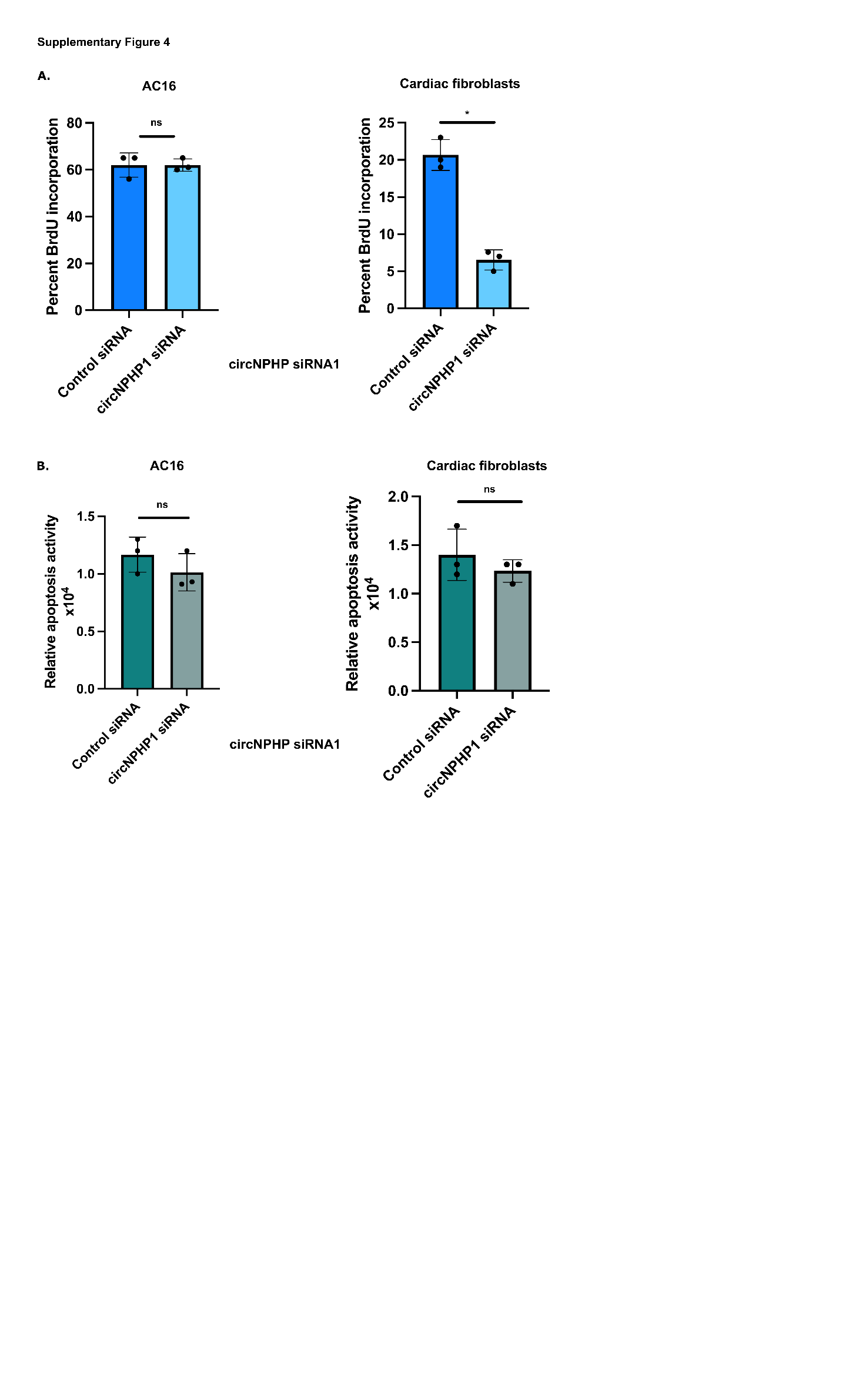
Supplementary Figure 4**

**Supplementary Figure 4: CircNPHP1 regulates cellular proliferation in cardiac fibroblasts**

(A) AC16 (left panel) and cardiac fibroblasts (right panel) were transfected with 30nM of circNPHP1 siRNA or control siRNA. 48 hours post transfection, cells were harvested for proliferation assay (BrDU incorporation) **(N=**3) and (B) apoptosis assay (**N=3)**. Data are expressed as mean*±*SEM and were assessed by unpaired Student *t* test. *P<0.05, **P<0.01, ***P<0.001, and ns = not significant. Biological replicates in AC16 and cardiac fibroblast are carried out on cells from different aliquots and passages of the same lot.

**Supplementary Figure 5**

**
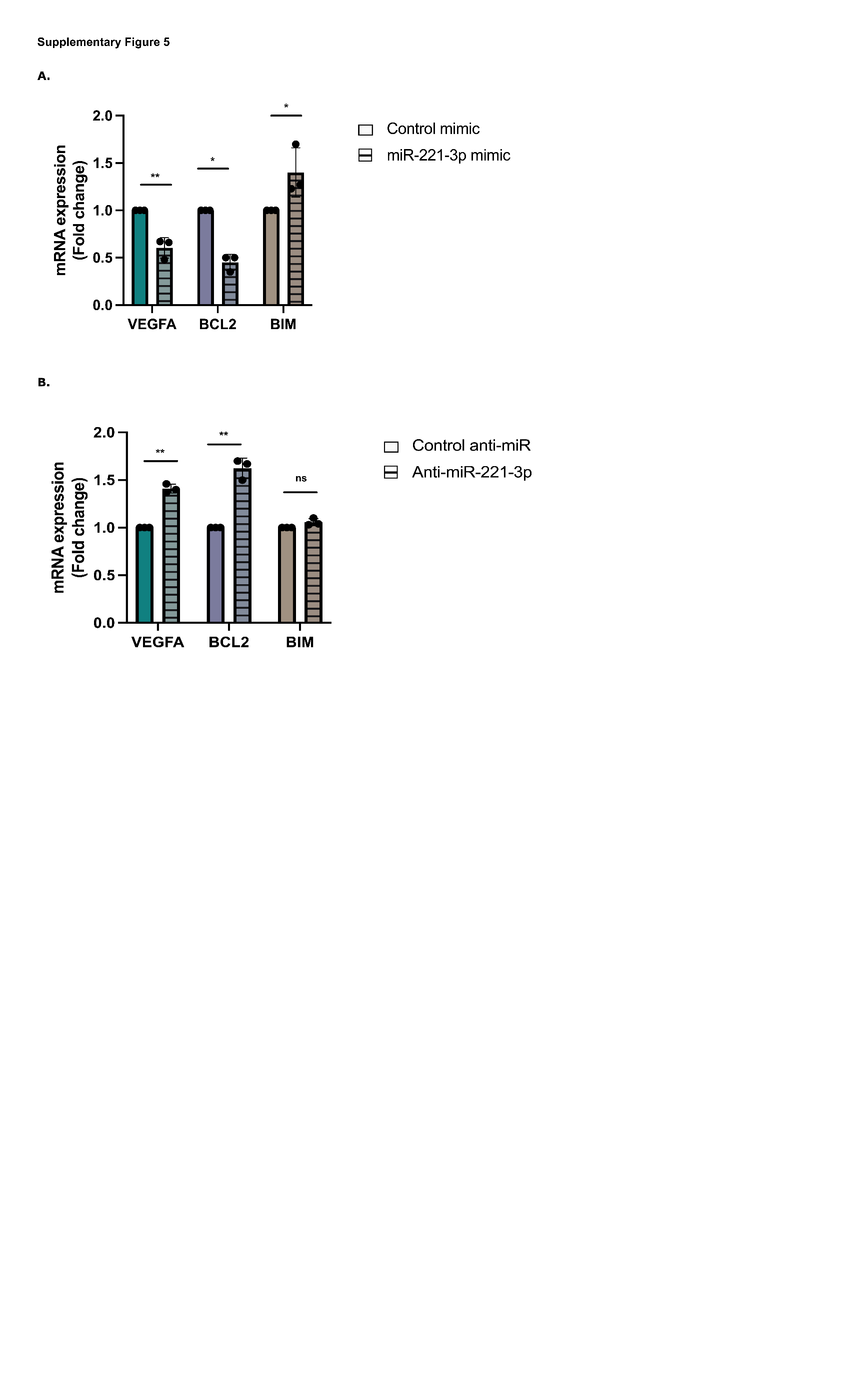
**

**Supplementary Figure 5: miR-221-3p regulates VEGFA and BCL2**

(A) HUVECs were transfected with 30nM of miR-221-3p mimic or control mimic (B) anti-miR-221-3p or anti-miRNA negative control. 48 hours post transfection, cells were harvested for qRT-PCR of BIM, BCL2 and VEGFA. Fold change in mRNA expression is relative to control (control mimic (A) or anti-miRNA negative control (B); 18S is used as housekeeping gene; N=3. Data are expressed as mean*±*SEM and were assessed by unpaired Student *t* test between the two groups. *P<0.05, **P<0.01, ***P<0.001, and ns = not significant.

**
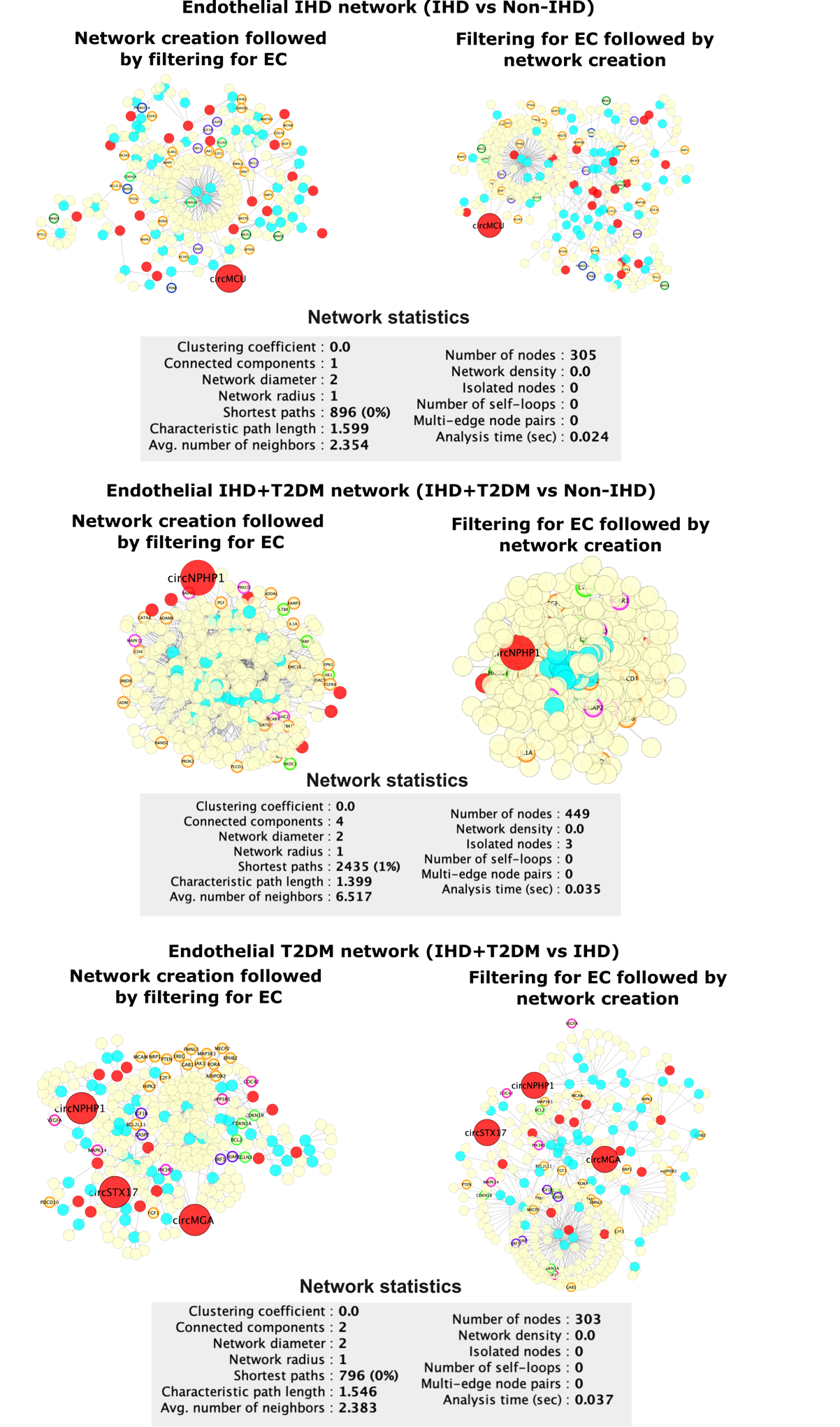
Supplementary Figure 6**

**Supplementary Figure 6: Networks created before and after filtering for RNA expression in ECs**

To show that the network structure, number of nodes and various parameters remain unchanged irrespective of filtering of the nodes (RNAs) based on EC expression (using GEO datasets; outlined in methods). The networks on the left indicate the structure and nodes obtained after creating the networks followed by filtering the nodes for EC expression. The networks on the right indicate the structure and nodes obtained by filtering nodes based on EC expression first followed by network creation. As indicated, the various parameters of the network remain unchanged as do the nodes.

**Supplementary Table 1**

**KEY:**

**Sex:** M-Male; F-Female

**LV function**

1 - Normal LV function (>55%)

2 - Moderate LV impairment (>30% and <55%)

3 - Poor LV function (<30%)

***Oral antidiabetics**

Metformin (1 gm once a day or 1 gm twice a day = max daily - 2gms-/day)

**Supplementary Table 1: Patient characteristics cohort 1**

Table shows median values for continuous variables and percentages for categorical variables.

**Supplementary Table 2**

**KEY:**

**Sex:** M-Male; F-Female

**LV function**

1 - Normal LV function (>55%)

2 - Moderate LV impairment (>30% and <55%)

3 - Poor LV function (<30%)

***Oral antidiabetics**

Metformin (1 gm once a day or 1 gm twice a day = max daily - 2gms-/day)

**Supplementary Table 2: Patient characteristics cohort 2**

Table shows median values for continuous variables and percentages for categorical variables.

**Supplementary Table 3**

**
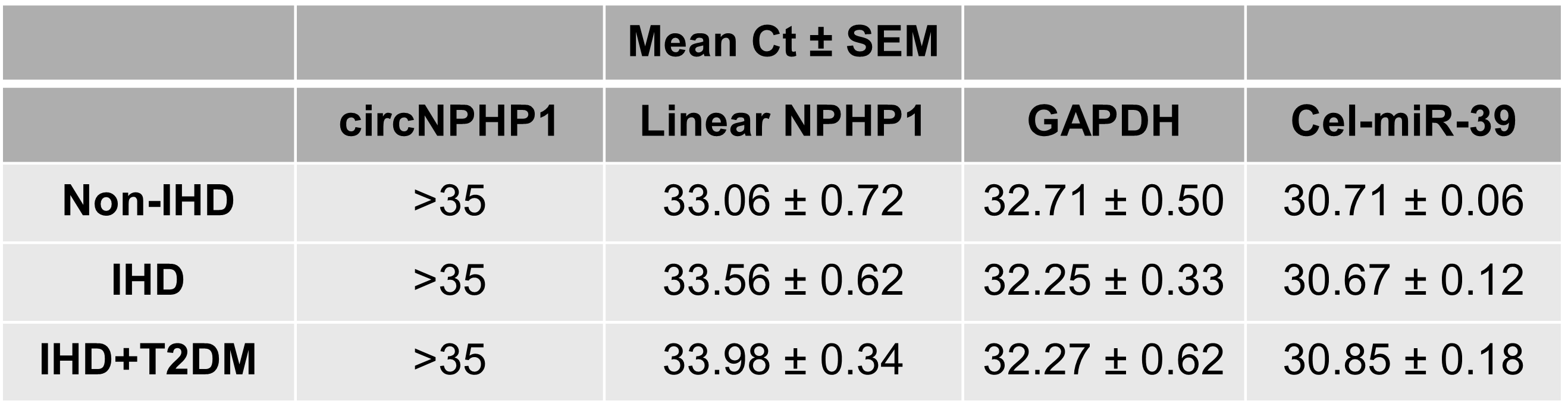
**

**Supplementary Table 3: Quantification of the expression levels of CircNPHP1 and linear NPHP1 in the plasma of patients**

qRT-PCR analysis of circNPHP1 and linear NPHP1 of patient plasma from non-IHD (N=5), IHD (N=6), IHD+T2DM (N=4). GAPDH is used as endogenous control and cel-miR-39 as spike-in control.

The table shows the average Ct values ± SEM. Ct values > 35 were considered undetectable.
