# Supplementary material for: Human Left Ventricle circRNA-miRNA-mRNA Network Analyses Reveal a Novel Proangiogenic Role for circNPHP1 Under Ischemic Conditions
Source: JACC Basic Transl Sci. 2026 Jan 20;11(2):101468. doi: 10.1016/j.jacbts.2025.101468 (PMC12859194; doi:10.1016/j.jacbts.2025.101468)
Supplement: Supplementary File 3 [file mmc4.pdf]

Supplementary File 3: Resources Table

Antibodies

Target antigen	Vendor or Source	Catalog #	Working concentration	Lot # (preferred but not required)	Persistent ID / URL
VEGF-A (WB)	Abcam	ab46154	1:2000	1051635-1	https://www.abcam.com/products/primary-antibodies/vegfa-antibody-ab46154.html
BCL2 (WB)	Cell Signalling Technology	3498S	1:1000	6	https://www.cellsignal.com/products/primary-antibodies/bcl-2-d17c4-rabbit-mab/3498
Lamin B1 (WB)	Cell Signalling Technology	12586S	1:1000	2	https://www.cellsignal.com/products/primary-antibodies/lamin-b1-d4q4z-rabbit-mab/12586
Mouse-anti-rabbit IgG-HRP (WB)	Santa Cruz	sc-2357	1:2000	H2218	https://www.scbt.com/p/mouse-anti-rabbit-igg-hrp
Phalloidin-FITC (Matrigel assay)	ThermoFisher Scientific	A12379	1:2500		https:// www.thermofisher.com/order/ catalog/product/A12379

Cultured Cells

Name	Vendor or Source	Persistent ID / URL
Human Umbilical Vein Endothelial Cells 2 (HUVEC 2) (pooled donors, cryopreserved vials)	Promocell	Catalogue no: C-12208<br>https://promocell.com/product/human-umbilical-vein-endothelial-cells-2-huvec-2/<br><br>Lot numbers: 447Z004, 450Z015, 466Z022, 503Z026
Human Cardiac Microvascular Endothelial Cells (HCMEC) (single donors, cryopreserved vials)	Promocell	Catalogue no: C-12285<br>https://promocell.com/product/human-cardiac-microvascular-endothelial-cells-hcmec/<br><br>Lot numbers: 440Z021.5, 440Z021.4, 446Z001.1, 492Z009.4
Human Cardiac Fibroblasts (HCF), unknown if pooled or single donor cryopreserved vials	Promocell	Catalogue no: C-12375<br>https://promocell.com/product/human-cardiac-fibroblasts-hcf/<br><br>Lot number: 491Z026.1
Human ventricular cardiomyocyte (AC-16 cells)	Donated by collaborator (Dr Rajesh Katare University of Otago)	https://www.sigmaaldrich.com/GB/en/product/mm/scc109

Data Availability

Description	Source / Repository	Persistent ID / URL
LV biopsies whole transcriptome RNA sequencing	GEO/SRA database	PRJNA1336212
LV biopsies small RNA sequencing	GEO/SRA database	PRJNA1336212
Single cell sequencing data of Human Heart Atlas	The European Nucleotide Archive (ENA) database<br><br>Processed data exploration from Heart Cell Atlas v2 website	ERP123138<br><br>https://www.heartcellatlas.org

Whole transcriptome bulk RNA sequencing of different cells	GEO/SRA database	PRJNA1336212
RNA sequencing data on circular RNAs (HUAECs)	GEO database	GSE100242
Expression profiling of miRNAs (HCAECs) by RT-PCR	GEO database	GSE53315
Transcriptomic profiling by RNA-seq (HCAECs)	GEO database	GSE134489

List of oligos

Description	Sequence	Vendor	Persistent ID / URL
Control siRNA	ON-TARGETplus Non-targeting Pool	Horizon Discovery Catalog# D-001810-10-05	https://horizondiscovery.com/en/gene-modulation/knockdown/sirna/products/on-targetplus-sirna-reagents
CircNPHP1 siRNA1	Sense: 5'AGAAGGCACUAUAAAC ACUGUUU 3' Antisense: 5'ACAGUGUUUAUAGUGC CUUCUUU 3'	Horizon Discovery (Custom made)	-
CircNPHP1 siRNA2	Sense: 5'ACAGAAGGCACUAUAA ACACUUU 3' Antisense: 5'AGUGUUUAUAGUGCCU UCUGUUU 3'	Horizon Discovery (Custom made)	-
Linear NPHP1 siRNA	See URL	Santa Cruz Biotechnology Catalog# sc-40769	https://www.scbt.com/p/nephrocystin-sirna-h-shrna-and-lentiviral-particle-gene-silencers
Human has-miR-221-3p (microRNA Mimic)	See URL	Horizon Discovery Catalog# C-300578-05-0010	https://horizondiscovery.com/en/gene-modulation/knockdown/mirna/products/miridian-microrna-mimic?nodeid=mirnaprecursor-mi0000298
Mimic Negative Control #1	See URL	Horizon Discovery Catalog# CN-001000-01-05	https://horizondiscovery.com/en/gene-modulation/knockdown/controls/products/miridian-microrna-mimic-negative-control-1
Anti-miR™ miRNA Inhibitor miR-221-3p	See URL	ThermoFisher Scientific Catalog# AM17000	https:// www.thermofisher.com/order/ catalog/product/AM17000
Anti-miR™ miRNA inhibitor Negative Control #1	See URL	ThermoFisher Scientific Catalog# AM17010	https:// www.thermofisher.com/order/ catalog/product/AM17010
cel-miR-39 (microRNA (cel-miR-39) Spike-In Kit)	See URL	Norgen Biotek Catalog# SKU 59000	https://norgenbiotek.com/product/microrna-cel-mir-39-spike-kit
Control ASO Probe:	5’TGCGTAACGAACGACG AATCGTCGCAGATC-3’[Biotin-TEG]	Sigma-Aldrich (Custom made)	-

circNPHP1 ASO Probe1:	5'CTACAGAAGGCACTGATAAACACTGTTGAT-3'[Biotin-TEG]	Sigma-Aldrich (Custom made)	-
circNPHP1 ASO Probe2**:**	5'AGAAGGCACTATAAACACTGT-3'[Biotin-TEG]	Sigma-Aldrich (Custom made)	-
miRNA Control sequence:	**Antisense:** **5'**GAUGGCAUUCGAUCAGUUCUA-3'[Biotin] **Sense:** 5'UAGAACUGAUCGAAUGCCUAUC-3'	Sigma-Aldrich (Custom made)	-
miRNA 221-3p sequence:	**Antisense:** **5'**AGCUACAUUGUCUGCUGGGUUUC -3'[Biotin] **Sense:** 5'GAAACCCAGCAGACAAUGUAUGCU-3'	Sigma-Aldrich (Custom made)	-
miRNA 222-3p sequence:	**Antisense:** **5'**AGCUACAUCUGGCUACUGGGU -3'[Biotin] **Sense:** 5'ACCCAGUAGCCAGAUGUAUGCU -3'	Sigma-Aldrich (Custom made)	-
miRNA 139-5p sequence:	**Antisense:** **5'**UCUACAGUGCACGUGUCUCCAGU -3'[Biotin] **Sense:** 5'ACUGGAGACACGUGCACUGUAGA -3'	Sigma-Aldrich (Custom made)	-

List of qRT-PCR primers

**Description**	**Sequence**	**Vendor**
Linear NPHP1	Forward 5' CTGCCACTGTCACACTGCATTC 3'<br>Reverse 5' GTTTACCATGACTGCGTGCTCC 3'	Sigma-Aldrich
CircNPHP1	Forward 5' TCTTACAACCAGAGCTCATGCC 3'<br>Reverse 5' AAGCTGTGAGAGCGTGGAA	Sigma-Aldrich
VEGFA	Forward 5' AGAGCAAGACAAGAAAATCC 3'<br>Reverse 5' TACAAACAAATGCTTTCTCC 3'	Sigma-Aldrich
BCL2	Forward 5' ACTGGAGAGTGCTGAAGATTG 3'<br>Reverse 5' AGTCTACTTCCTCTGTGATGTTG 3'	Sigma-Aldrich
BCL2L11 (BIM)	Forward 5' GGCCCCTACCTCCCTACA 3'<br>Reverse 5' GGGGTTTGTGTTGATTTGTCA 3'	Sigma-Aldrich

18s	Forward 5' CCCAGTAAGTGCGGGTCAT 3'<br>Reverse 5' CCGAGGGCCTCACTAAACC 3'	Sigma-Aldrich
GAPDH	Forward 5' GACTCATGACCACAGTCCATGC 3'<br>Reverse 5' AGAGGCAGGGATGATGTTCTG 3'	Sigma-Aldrich
cel-miR-39	Forward 5' TTGCAGCTCTCATAGAAGGAACCG3′<br>Reverse 5′-GTTTCAGCCGAGACTAGACTTTGAGC3′	Sigma-Aldrich
CircMCU	Forward 5'CTGTTCACGCAGGGGAAACT 3'<br>Reverse 5' AGCAGCTAAGATGTCACTGGC 3'	Sigma-Aldrich
CircSTX17	Forward 5' ATGCTGCAGAATCGTGGGAA 3'<br>Reverse 5' TCTGAGAACTAGCTTCAGCTTCA 3'	Sigma-Aldrich
CircMGA	Forward 5' TGTAAGCCCTGGGAGTACCT 3'<br>Reverse 5' TCTGGTCTAACGGTGAGGCT3'	Sigma-Aldrich
hsa-miR-221-3p	hsa-miR-221-3p miRCURY LNA miRNA PCR Assay (predesigned) GeneGlobe ID - YP00204532	Qiagen<br>Catalog# 339306
hsa-miR-222-3p	hsa-miR-222-3p miRCURY LNA miRNA PCR Assay (predesigned) GeneGlobe ID - YP00204551	Qiagen<br>Catalog# 339306
hsa-miR-299-3p	hsa-miR-299-3p miRCURY LNA miRNA PCR Assay (predesigned) GeneGlobe ID - YP00204702	Qiagen<br>Catalog# 339306
hsa-miR-139-3p	hsa-miR-139-3p miRCURY LNA miRNA PCR Assay (predesigned) GeneGlobe ID - YP00205661	Qiagen<br>Catalog# 339306
hsa-miR-141-3p	hsa-miR-141-3p miRCURY LNA miRNA PCR Assay (predesigned) GeneGlobe ID - YP00204504	Qiagen<br>Catalog# 339306
U6	U6 snRNA (v2) miRCURY LNA miRNA PCR Assay (predesigned) GeneGlobe ID - YP00204532	Qiagen<br>Catalog# 339306

Others

Description	Source / Repository	Persistent ID / URL
Endothelial cell growth medium 2	Promocell	https://promocell.com/product/endothelial-cell-growth-medium-2/
Endothelial cell growth medium MV	Promocell	https://promocell.com/product/endothelial-cell-growth-medium-mv/
Fibroblast Growth Medium 3	Promocell	https://promocell.com/product/fibroblast-growth-medium-3/
DMEM-F12	ThermoFisher Scientific	https://www.thermofisher.com/order/catalog/product/10565018
Lipofectamine^{TM} 2000	ThermoFisher Scientific	https://www.thermofisher.com/order/catalog/product/11668027

BrdU Cell Proliferation ELISA kit	Abcam	https://www.abcam.com/products/elisa-kits/brdu-cell-proliferation-elisa-kit-colorimetric-ab126556.html
Growth Factor Reduced Matrigel	Corning	https://ecatalog.corning.com/life-sciences/b2b/DK/en/Surfaces/Extracellular-Matrices-ECMs/Corning%C2%AE-Matrigel%C2%AE-Matrix/p/356231
RealTime-Glo™ Annexin V Apoptosis and Necrosis Assay kit	Promega	https://www.promega.co.uk/products/cell-health-assays/apoptosis-assays/realtime-glo-annexin-v-apoptosis-assay/?catNum=JA1011
Streptavidin Dynabeads	New England Biolabs	https://www.neb.com/en-gb/products/s1420-streptavidin-magnetic-beads
RNase R enzyme	New England Biolabs	https://www.neb.com/en-gb/products/m0100-rnase-r?srsltid=AfmBOopayLqzuMWgtNf_3OKBwn_iZbKdy9ZLbAg5B7aejvBQniKKiNgy
miRNeasy mini kit	Qiagen	https://www.qiagen.com/us/products/discovery-and-translational-research/dna-rna-purification/rna-purification/mirna/mirneasy-kits?catno=217004
QIAzol reagent	Qiagen	https://www.qiagen.com/us/products/discovery-and-translational-research/lab-essentials/buffers-reagents/qiazol-lysis-reagent
miRNeasy Serum/Plasma Kit	Qiagen	https://www.qiagen.com/us/products/discovery-and-translational-research/dna-rna-purification/rna-purification/mirna/mirneasy-serumplasma-kit
PrimeScript RT-PCR kit	TakaraBio	https://www.takarabio.com/products/real-time-pcr/reverse-transcription-prior-to-qpcr/convenient-master-mix-for-real-time-pcr
TB Green Premix Ex Taq II Kit	TakaraBio	https://www.takarabio.com/products/real-time-pcr/real-time-pcr-kits/dye-based-qpcr-mixes/tb-green-premix-ex-taq-ii-(tli-rnase-h-plus)?catalog=RR82LR
miRCURY LNA RT Kit	Qiagen	https://www.qiagen.com/us/products/discovery-and-translational-research/pcr-qpcr-dpcr/qpcr-assays-and-instruments/mirna-qpcr-assay-and-panels/mircury-lna-rt-kit
miRCURY LNA SYBR Green PCR Kit	Qiagen	https://www.qiagen.com/us/products/discovery-and-translational-research/pcr-qpcr-dpcr/qpcr-assays-and-instruments/mirna-qpcr-assay-and-panels/mircury-lna-sybr-green-pcr-kits
Immobilon Crescendo Western HRP	Merck	https://www.merckmillipore.com/GB/en/product/Immobilon-Crescendo-Western-HRP-substrate-100-mL,MM_NF-WBLUR0100
Protease inhibitor cocktail	Merck	https://www.sigmaaldrich.com/GB/en/product/roche/11836153001?utm_source=google&utm_medium=cpc&utm_campaign=15001183107&utm_content=127306761543&gclid=CjwKCAjwwr6wBhBcEiwAfMEQsyfkKcrb8eE7ZpKtmtuk13a6ja4dLQZOrlOxIenI4CT6Y6NEL0S5txoC7ZQQAvD_BwE
RIPA buffer	Sigma-Aldrich	https://www.sigmaaldrich.com/GB/en/product/sigma/r0278
*mir*Vana kit (AM1560)	ThermoFisher Scientific	https://www.thermofisher.com/order/catalog/product/AM1560?gclid=CjwKCAjwwr6wBhBcEiwAfMEQsw4Pcovfnp_wS2LJKczpGy-I84MvofdhpFVR95-G-g11aQJ4QnHF0hoC2KYQAvD_BwE&ef_id=CjwKCAjwwr6wBhBcEiwAfMEQsw4Pcovfnp_wS2LJKczpGy-I84MvofdhpFVR95-G-g11aQJ4QnHF0hoC2KYQAvD_BwE:G:s&s_kwcid=AL!3652!3!606515189508!!!g!!!16893189581!143415109412&cid=bid_sap_rst_r01_co_cp0000_pjt0000_bid00000_0se_gaw_dy_awa_con&gad_source=1

DNA-*free* DNA removal kit	Fisher Scientific	https://www.fishersci.com/shop/products/ambion-dna-i-free-i-dna-removal-kit-1/AM1906
NEBNext rRNA depletion kit	New England Biolabs	https://www.neb.com/en-gb/products/e6350-nebnext-rrna-depletion-kit-human-mouse-rat-with-sample-purification-beads
NEBNext Ultra II Directional RNA Library Prep Kit for Illumina	New England Biolabs	https://www.neb.com/en-gb/products/e7760-nebnext-ultra-ii-directional-rna-library-prep-kit-for-illumina
